# Defining Global Gene Expression Changes of the Hypothalamic-Pituitary-Gonadal Axis in Female sGnRH-Antisense Transgenic Common Carp (*Cyprinus carpio*)

**DOI:** 10.1371/journal.pone.0021057

**Published:** 2011-06-10

**Authors:** Jing Xu, Wei Huang, Chengrong Zhong, Daji Luo, Shuangfei Li, Zuoyan Zhu, Wei Hu

**Affiliations:** 1 State Key Laboratory of Freshwater Ecology and Biotechnology, Institute of Hydrobiology, Chinese Academy of Sciences, Wuhan, China; 2 Graduate School of the Chinese Academy of Sciences, Beijing, China; Temasek Life Sciences Laboratory, Singapore

## Abstract

**Background:**

The hypothalamic-pituitary-gonadal (HPG) axis is critical in the development and regulation of reproduction in fish. The inhibition of neuropeptide gonadotropin-releasing hormone (GnRH) expression may diminish or severely hamper gonadal development due to it being the key regulator of the axis, and then provide a model for the comprehensive study of the expression patterns of genes with respect to the fish reproductive system.

**Methodology/Principal Findings:**

In a previous study we injected 342 fertilized eggs from the common carp (*Cyprinus carpio*) with a gene construct that expressed antisense sGnRH. Four years later, we found a total of 38 transgenic fish with abnormal or missing gonads. From this group we selected the 12 sterile females with abnormal ovaries in which we combined suppression subtractive hybridization (SSH) and cDNA microarray analysis to define changes in gene expression of the HPG axis in the present study. As a result, nine, 28, and 212 genes were separately identified as being differentially expressed in hypothalamus, pituitary, and ovary, of which 87 genes were novel. The number of down- and up-regulated genes was five and four (hypothalamus), 16 and 12 (pituitary), 119 and 93 (ovary), respectively. Functional analyses showed that these genes involved in several biological processes, such as biosynthesis, organogenesis, metabolism pathways, immune systems, transport links, and apoptosis. Within these categories, significant genes for neuropeptides, gonadotropins, metabolic, oogenesis and inflammatory factors were identified.

**Conclusions/Significance:**

This study indicated the progressive scaling-up effect of hypothalamic sGnRH antisense on the pituitary and ovary receptors of female carp and provided comprehensive data with respect to global changes in gene expression throughout the HPG signaling pathway, contributing towards improving our understanding of the molecular mechanisms and regulative pathways in the reproductive system of teleost fish.

## Introduction

The hypothalamic-pituitary-gonadal (HPG) axis is critical in the development and regulation of the reproductive, endocrine, and immune systems, in fish and other vertebrates [1]. The neuropeptide gonadotropin-releasing hormone (GnRH), which is secreted from the hypothalamus, is often described as the key regulator of this significant axis pathway [2]. Earlier observations have established that multiple forms of GnRH have the capacity to activate anterior pituitary receptors to stimulate the expression and release of reproductive hormones into the blood, including luteinizing hormone (LH), follicle-stimulating hormone (FSH) and growth hormone (GH). As a result, oogenesis and/or spermatogenesis processes are initiated, as well as sex steroid and inhibin production [2]–[6]. Recent studies in zerbafish (*Danio rerio*), medaka (*Oryzias latipes*) and goldfish (*Carassius auratus*) reported separately that some neuropeptides and neurotransmitters could interact with GnRH in the control of pituitary hormone release [7]–[9]. These results lead us to hypothesize that there may be a number of factors involved in the HPG signaling pathway that reflect the complexity of the teleost reproductive system.

In the common carp (*Cyprinus carpio*), which belongs to the family of Cyprinidae, two GnRH variants have been isolated and shown to be the same as GnRH-II (chicken GnRH-II, cGnRH-II) and GnRH-III (salmon GnRH, sGnRH), respectively. As in other teleosts, the two forms in common carp have different tissue distributions, expression levels, and regulation modes [10], [11]. As in plants and mammals, the antisense transgenic technique has been applied in fish to determine the effectiveness of antisense and whether the inhibition of GnRHs is an effective tool to induce sterility [12]–[14]. Uzbekova et al. inhibited endogenous sGnRH expression by a recombinant vector containing antisense sGnRH cDNA in Atlantic salmon (*Salmo salar*) [12]. Maclean et al. obtained tilapia that were sterile or had very low fertility using the antisense transgenic technique [13]. Hu et al. [14] found that when sGnRH expression was inhibited by sGnRH antisense in common carp, the plasma LH (previously called gonadotropin-II or GtH-II) level in the males was significantly reduced, subsequently diminishing or severely limiting gonad development. As a result, this study generated some transgenic carp with abnormal gonad tissues. In the present study, we have begun to examine whether these fish might be a model to study changes in gene expression throughout the HPG axis with respect to fish reproduction.

The molecular techniques of suppressive subtractive hybridization (SSH) and microarray, have been preferentially used in recent studies as a high throughout screening approach for differentially expressed genes in different developmental or tissue samples. In the 1990s, an SSH library was constructed by Blázquez to identify candidate genes involved in the control of reproduction in the pituitary [15]. Later, SSH and microarray were combined and considered to have an advantage in generating an equalized representation of differentially expressed ESTs, irrespective of the relatively disproportionate concentrations of the transcripts, hence guaranteeing the identification of the differentially expressed genes. For example, Villaret et al. [16] reported that 13 independent genes were significantly overexpressed in human tumor tissues in comparison with normal tissue when using subtractive and microarray technology. Similarly, 240 genes were identified as important in the development and maturation of the rainbow trout (*Oncorhynchus mykiss*) ovary, when using subtractive ovary and testis libraries and microarray analyses [17]. Furthermore, Vallée et al. [18] have reported that combined SSH and microarray techniques have even been utilized in three different vertebrate species (bovine, mouse, and frog), to identify the novel oocyte-specific genes.

In the present study, we used SSH combined with cDNA microarray analysis to screen for the changes in gene expression of the HPG axis between the female sGnRH-antisense transgenic and control common carp. Furthermore, we analyzed the biological processes associated with the activity of these reproductive system genes. In the case of some differentially expressed genes, real-time PCR was used to validate their differences. We hypothesize that differential expression patterns may account for the complex regulatory mechanisms of the reproductive system in teleost fish.

## Results

### The developmental status and histological characterization of the transgenic carp ovary

The transgenic common carp were generated by the injection of sGnRH antisense RNA and evaluated by tail clip PCR. Carp retaining the transgene were named AS(+) and totaled 102. Their gonadal development was checked in the next four breeding seasons. As shown in Table S1, 64 of the 102 carp had normal gonads with sperm or eggs. The other 38 carp had abnormal gonads, of which 14 were male carp, 12 were female carp, and 12 had no gonad tissues.

**Figure 1:**
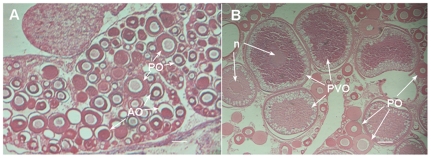
Histological sections of four-year-old AS(+) and control fish. (A) abnormally developed ovary section of AS(+) carp. (B) normally developed ovary section of control carp. The scale bar = 100 µm; n, nuclear; PO, primary oocyte; PVO, previtellogenic oocyte; AO, atretic oocyte.

Twelve four-year-old female AS(+) carp with abnormal ovaries were selected and dissected. The sGnRH antisense RNA expression was confirmed in genomic DNA and hypothalamic cDNA (Shown in Figure S1). In comparison with the complete ovary tissues of normally developed control carp at the same age, the ovaries of AS(+) carp were found to be unilateral and undeveloped (Shown in Figure S2). Observation of histological sections under the microscope showed that AS(+) ovaries were pink, containing some atretic oocytes and primary oocytes, that were polygonal round or elliptic with a concentrated nucleus (Figure 1A). By contrast, control ovaries of the normal carp were creamy yellow in color, and contained a large number of oocytes at different developmental stages (Figure 1B). These results implied that the ovary of sGnRH-antisense transgenic carp AS(+) did not completely develop, being restricted in the early stages of development.

### Suppressive subtractive hybridization and microarray hybridization

To identify the genes involved in HPG axis pathway differentially expressed between the transgenic and the control carp, six sublibraries of the hypothalami, pituitaries, and ovaries were created using SSH as a technique. From the six sublibraries, 15,998 single-insert clones were selected for construction of microarray cDNA chips. The hybridizations were then applied with cDNA probes labeled with fluorochromes. An example of one microarray hybridization is shown in Figure S3.

**Figure 2:**
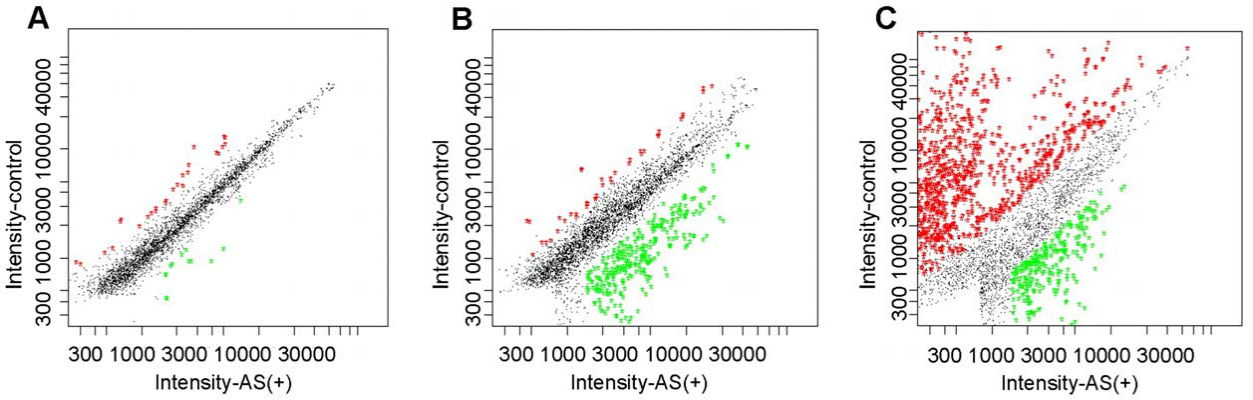
Scatterplot charts of the duplicate spot intensities obtained from cDNA microarray analyses. Cy3 and Cy5 dyes were used to label cDNA probes of the hypothalamus (A), pituitary (B), and ovary (C) prepared from AS(+) and control carp. The X coordinate value is the gene expression level (intensity value) in the test with the transgenic-Cy3 cDNA probes. The Y coordinate value is the other test with the control-Cy5 cDNA probes. Each dot represents a duplicate clone, and the green and red dots represent the clones up-regulated in AS(+) and control carp, respectively. The black dots indicate the clones with few changes between them.

The duplicate spot intensities of the 15,998 cDNA spots for transgenic and control carp are presented as scatter plot charts, derived from Imagine software. In the scatter plot charts, the X coordinate value is the gene expression level (intensity value) in the test with the transgenic-Cy3 cDNA probes, and the Y coordinate value is the other test with the control-Cy5 cDNA probes. The green and red dots represent the clones up-regulated in AS(+) and control carp, respectively. The black dots indicate the clones with few changes between them (Figure 2). A total of 1,064 differentially expressed clones (FDR <0.01 and fold change ≥2) were obtained from the AS(+) using signal treatment. These include 23 clones from the hypothalamus, 198 from the pituitary, and 843 from the ovary. Additionally, the number of down-regulated and up-regulated clones in the three tissues was 16 and seven (hypothalamus), 171 and 27 (pituitary), 684 and 159 (ovary), respectively (data not shown).

### Data analysis

The 1,064 differentially expressed clones obtained from the microarry hybridization were sequenced and analyzed in the National Center for Biotechnology Information (NCBI) for homology. After gene duplicate fragment checking, 249 valid ESTs were obtained, of which 162 ESTs had significant homology with known accessions GenBank database (*E* values ≤1e−5). This included 6 ESTs from the hypothalamus, 17 from the pituitary and 139 from the ovary. The remaining ESTs, about 87, were determined to be new, including 3 from the hypothalamus, 11 from the pituitary and 73 from the ovary. In additional, the confirmed amount of down-regulated and up-regulated ESTs of the three tissues were 5 and 4 for the hypothalamus, 16 and 12 for the pituitary, and 119 and 93 for the ovary, respectively. Details of each of the EST datasets are presented in Tables S2–S4. All of these ESTs have been submitted to NCBI and the GenBank Accession Numbers were JG017286-JG017531, JG390471-JG390473.

Of the ESTs explored for potential significant biological functions, 6 were expressed in the hypothalamus, and were found to be linked to the proteins arginine methyltransferase 1, hemoglobin alpha-globin, metallothionein II, Pro-melanin concentrating hormone, cytochrome oxidase subunit 1, and putative NADH dehydrogenase 5, respectively (Table 1). Of the pituitary level ESTs, 17 were primarily associated with hormones, metabolism and enzymes, such as gonadotropin subunits (gonadotropin common α subunit, FSHβ), growth hormone, cytochrome c oxidase, and NADH dehydrogenase (Table 2). At the gonad level, the main molecular functions of 139 known ESTs were involved in a range of physiological and regulatory biological processes. These included enzymes, inflammation and immune factors, oogenesis and ovulation factors, cellular component organization and assembly molecules, apoptosis molecules, and other functional molecules, such as cathepsin L, zona pellucida glycoproteins, tissue inhibitor of metalloproteinase 4, ovulatory protein-2 precursor and C-type lectin (Table 3).

**Table 1:** List of differentially expressed genes (FDR <0.01 and fold change ≥2) in AS(+) hypothalamus relative to control hypothalamus.

Category and gene identity (BlastX)	Homolog species	GenBank Accession No.	E-value	Microarray fold change AS(+)/control
Hemoglobin subunit alpha	*Cyprinus carpio*	P02016	3.00E-75	4.14
Arginine methyltransferase	*Danio rerio*	BAE97650	2.00E-89	2.27
Metallothionein II	*Cyprinus carpio*	AF249875	4.00E-81	0.5
MCH 1 precursor	*Oncorhynchus kisutch*	P56943	3.00E-21	0.49
Differentially expressed in malignant melanoma	*Homo sapiens*	AJ293391	2.00E-16	0.46
Cytochrome c oxidase subunit I	*Cyprinus carpio*	BAE97651	2.00E-47	0.39
NADH dehydrogenase	*Cyprinus carpio*	AP009047	3.00E-57	0.3

**Table 2 pone-0021057-t002:** List of differentially expressed genes (FDR <0.01 and fold change ≥2) in AS(+) pituitary relative to control pituitary.

Category and gene identity (BlastX)	Homolog species	GenBank Accession No.	E-value	Microarray fold change AS(+)/control
**Hormone**				
Gonadotropin beta subunit 1	*Cyprinus carpio*	X59888	e-141	0.47
Glycoprotein hormones alpha chain	*Cyprinus carpio*	P01221	7.00E-67	0.45
Growth hormone	*Carassius auratus*	ABY71031	1.00E-18	0.4
Secretogranin III	*Danio rerio*	NP_957051	6.00E-32	0.4
Brain aromatase	*Carassius auratus*	BAA23757	2.00E-82	0.48
**Metabolism**				
Cytochrome c oxidase subunit I	*Cyprinus carpio*	BAE97651	2.00E-47	0.46
Cytochrome c oxidase subunit II	*Cyprinus carpio*	ABX72174	2.00E-43	0.42
Cytochrome c oxidase subunit III	*Carassius auratus*	AAP38173	1.00E-68	0.43
NADH dehydrogenase	*Cyprinus carpio*	AP009047	3.00E-57	0.21
Hemoglobin subunit alpha	*Cyprinus carpio*	P02016	3.00E-75	4.42
**others**				
Arginine methyltransferase	*Danio rerio*	NM_200650	5.00E-35	2.37
DEAD (Asp-Glu-Ala-Asp) box polypeptide	*Danio rerio*	NP_998142	3.00E-72	4.23
Differentially expressed in malignant melanoma	*Homo sapiens*	AJ293391	2.00E-16	0.41
Pkm2 protein	*Danio rerio*	AAH67143	9.00E-30	2.13
Similar to melanoma inhibitory activity protein	*Danio rerio*	XP_001336607	4.00E-26	2.04

**Table 3:** List of differentially expressed genes (FDR <0.01 and fold change ≥2) in AS(+) ovary relative to control ovary.

Category and gene identity (BlastX)	Homolog species	GenBank Accession No	E-value	Microarray fold change AS(+)/control
**Hormone**				
Gonadotropin beta subunit 1	*Cyprinus carpio*	O13050	3.00E-63	0.26
Growth hormone	*Cyprinus carpio*	X51969	0	0.2
Isotocin precursor	*Cyprinus carpio*	AF322651	1.00E-18	3.94
MCH 1 precursor	*Oncorhynchus kisutch*	P56943	3.00E-21	0.32
**Metabolism**				
ATP synthase F0 subunit 6	*Ctenopharyngodon idella*	YP_001654971	1.00E-48	2.35
Cytochrome c oxidase subunit I	*Labeo batesii*	YP_913434	e-123	0.27
Cytochrome c oxidase subunit III	*Hemibarbus barbus*	YP_913282	2.00E-64	2.51
Cytochrome c oxidase subunit II	*Cyprinus carpio*	ABX72174	2.00E-43	3.06
Geminin	*Danio rerio*	NM_200086	9.00E-177	0.01
NADH dehydrogenase subunit 1	*Barbus barbus*	YP_913406	3.00E-57	2.16
NADH dehydrogenase subunit 2	*Cyprinus carpio*	BAE97650	9.00E-58	2.57
Superoxide dismutase	*Danio rerio*	XP_698633	2.00E-30	0.04
**Inflammation and immune**				
Basigin	*Danio rerio*	AAH56721	2.00E-63	2.42
C-type lectin	*Cyprinus carpio*	BAA95671	6.00E-80	0.01
Danio rerio high-mobility group box 1	*Danio rerio*	BC067193	e-105	2.7
Metallothionein-I	*Cyprinus carpio*	O13269	2.00E-10	0.21
Metallothionein II	*Cyprinus carpio*	AF249875	e-126	0.04
Pentraxin	*Cyprinus carpio*	BAB69039	8.00E-55	0.02
**Oogenesis and ovulation processes**				
Cathepsin L	*Lates calcarifer*	ABV59078	5.00E-37	0.03
Tissue inhibitor of metalloproteinases 4	*Takifugu rubripes*	AAO17737	7.00E-22	0.03
ZP2	*Cyprinus carpio*	CAA96572	e-141	0.01
ZP3	*Cyprinus carpio*	CAA88735	e-142	0.07

### Comprehensive analysis of the differentially expressed genes

**Figure 3:**
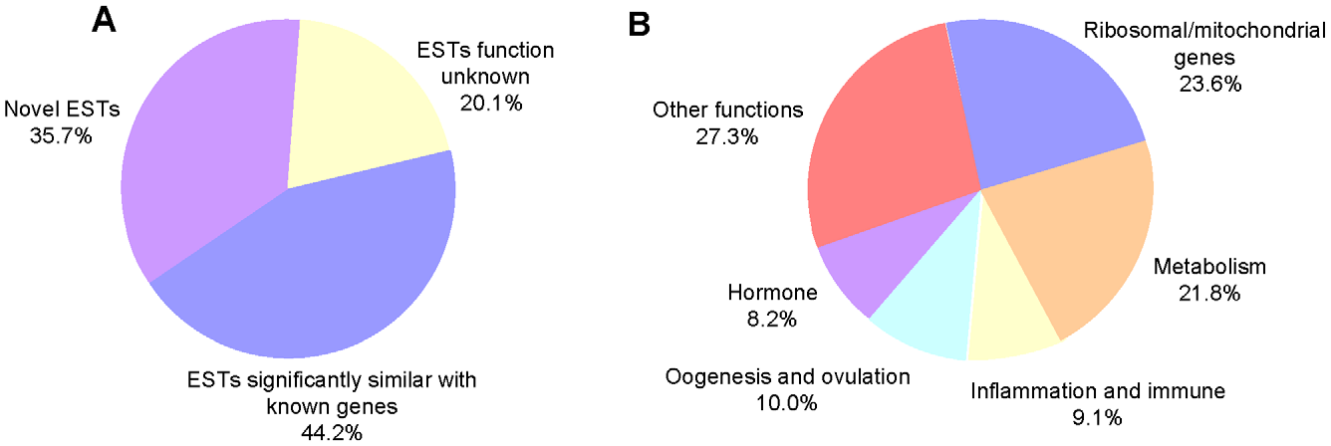
Pie diagram of the differentially expressed genes between AS(+) and control carp. The functional category was based on sequence homologies and Gene Ontology (GO) enrichment analyses. The percentages are shown under their categories. (A) Percentage of expressed sequence tags (ESTs) based on novel ESTs, ESTs significantly similar with known genes (*E* values ≤1e−5) and ESTs function unknown. (B) Function known ESTs were classified into ribosomal/mitochondrial genes, metabolism, inflammation and immune, oogenesis and ovulation, hormone, other molecular functions.

The functional category of the identified genes was determined based on sequence homologies and Gene Ontology (GO) enrichment analyses. The functional classifications showed that 10.4% were ribosomal/mitochondrial protein genes, 9.6% were related to protein, nucleic acid, or lipid metabolism, energy metabolism, or transport and/or signal transduction, 4.0% were related to inflammation and immune factors, 4.4% were related to oogenesis and ovulation processes, 3.6% were related to hormones, and 12% were related to other functions, such as binding proteins, ion transportation, cell signal transduction, and apoptosis. The function of 21.1% of genes was unknown, and 34.9% were termed novel ESTs as they bore no similarity to the known accessions of the GenBank database (Figure 3). Statistical analysis showed that 140 of these identified genes were down-regulated, while 109 were up-regulated.

**Figure 4:**
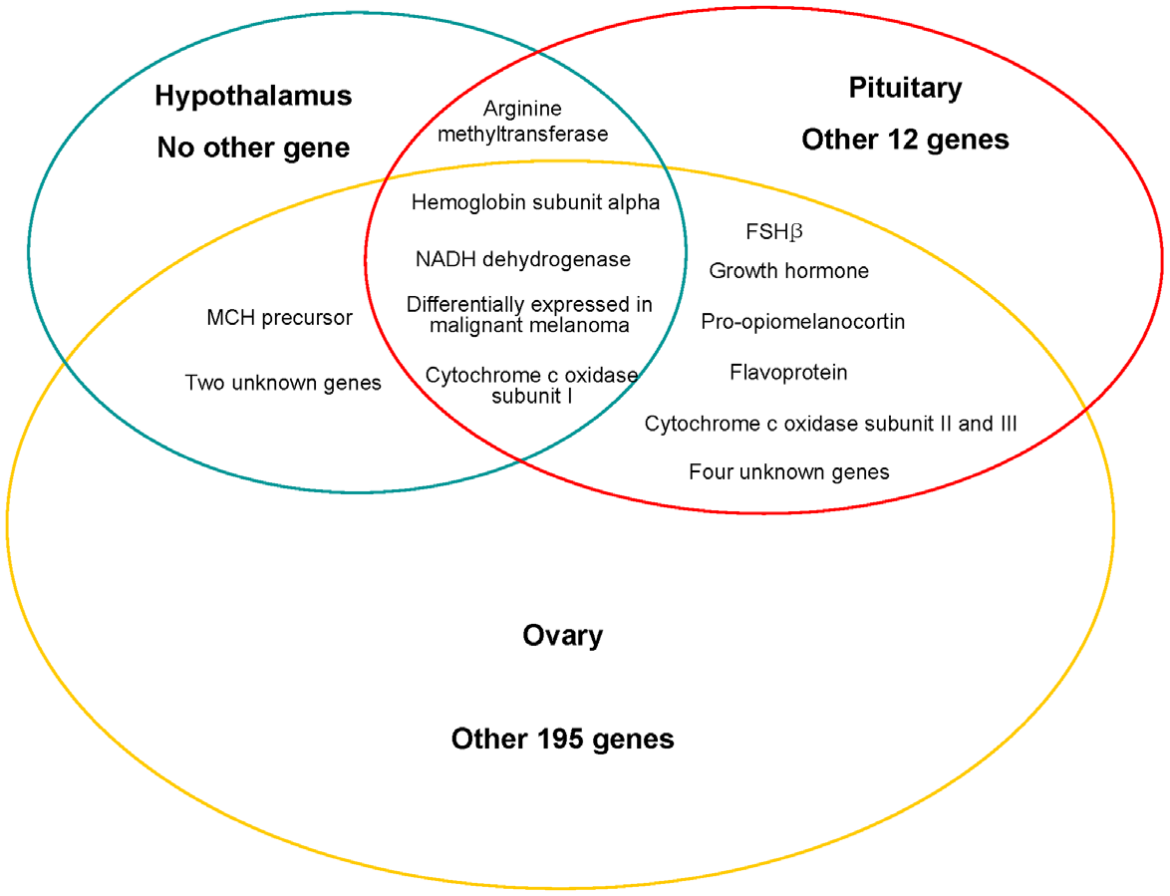
Schematic diagram comparing the differentially expressed genes of the hypothalamus, pituitary, and ovary. Cytochrome c oxidase subunit I, NADH dehydrogenase subunit I, hemoglobin subunit alpha, and an unknown factor homologous to a *Homo sapiens* mRNA differentially expressed in malignant melanoma were exhibited in all three libraries, flavoprotein, follicle stimulating hormone beta-subunit (FSHβ), pro-opiomelanocortin, growth hormone (GH), DEAD (Asp-Glu-Ala-Asp) box polypeptide, cytochrome c oxidase subunit II, and III, and four unknown factors were identified in the pituitary and ovary libraries. Melanin-concentrating hormone precursor (MCH) gene was exhibited in the hypothalamus and ovary libraries.

Four of these genes were exhibited in all three libraries (i.e. hypothalamus, pituitary, and ovary), comprising cytochrome c oxidase subunit I, NADH dehydrogenase subunit I, hemoglobin subunit alpha, and an unknown factor homologous to a *Homo sapiens* mRNA differentially expressed in malignant melanoma (Figure 4). Furthermore, the cytochrome c oxidase subunit I and the unknown melanoma factor were found to be down-regulated while hemoglobin subunit alpha was up-regulated in all three tissue libraries. NADH dehydrogenase subunit I was down-regulated only in the hypothalamus and pituitary libraries (Tables 1–3).

Meanwhile, 11 genes were identified in the pituitary and ovary libraries, namely flavoprotein (*fp*), FSHβ, pro-opiomelanocortin, GH, DEAD (Asp-Glu-Ala-Asp) box polypeptide, cytochrome c oxidase subunit II, and III, and four unknown factors (Figure 4). Of these, *fp* was up-regulated in two libraries, while GH and FSHβ were down-regulated. Additionally, the MCH precursor gene (*mch*) was strongly down-regulated in the hypothalamus and ovary of AS(+) carp (Tables 1–3).

### Validation of differentially expressed genes by real-time RT-PCR

To confirm the results obtained using combined SSH and microarray analyses, 25 candidates were selected from the six categories of GO enrichment analyses and amplified by real-time RT-PCR from three additional AS(+) and control carp samples. Among these genes, sGnRH, GH, gonadotropin beta subunit 1, and secretogranin III were associated with hormone functions. NADH dehydrogenase, superoxide dismutase, geminin, hemoglobin alpha, and a ribosomal gene were metabolic related factors. ZP2 and ZP3 were involved in oogenesis and ovulation directly. C-type lectin, pentraxin, basigin, and high-mobility group box 1 were inflammatory factors. MCH 1 precursor, melanoma inhibitory activity protein, S100 calcium binding protein A1, and GABA neurotransmitter transporter 1 were neuropeptides or factors associated with signal transduction. Others were arginine methyltransferase, pyruvate kinase M2, DEAD box polypeptide, B-cell translocation gene 4, and cystatin precursor. *β-actin* gene was used as control.

**Figure 5:**
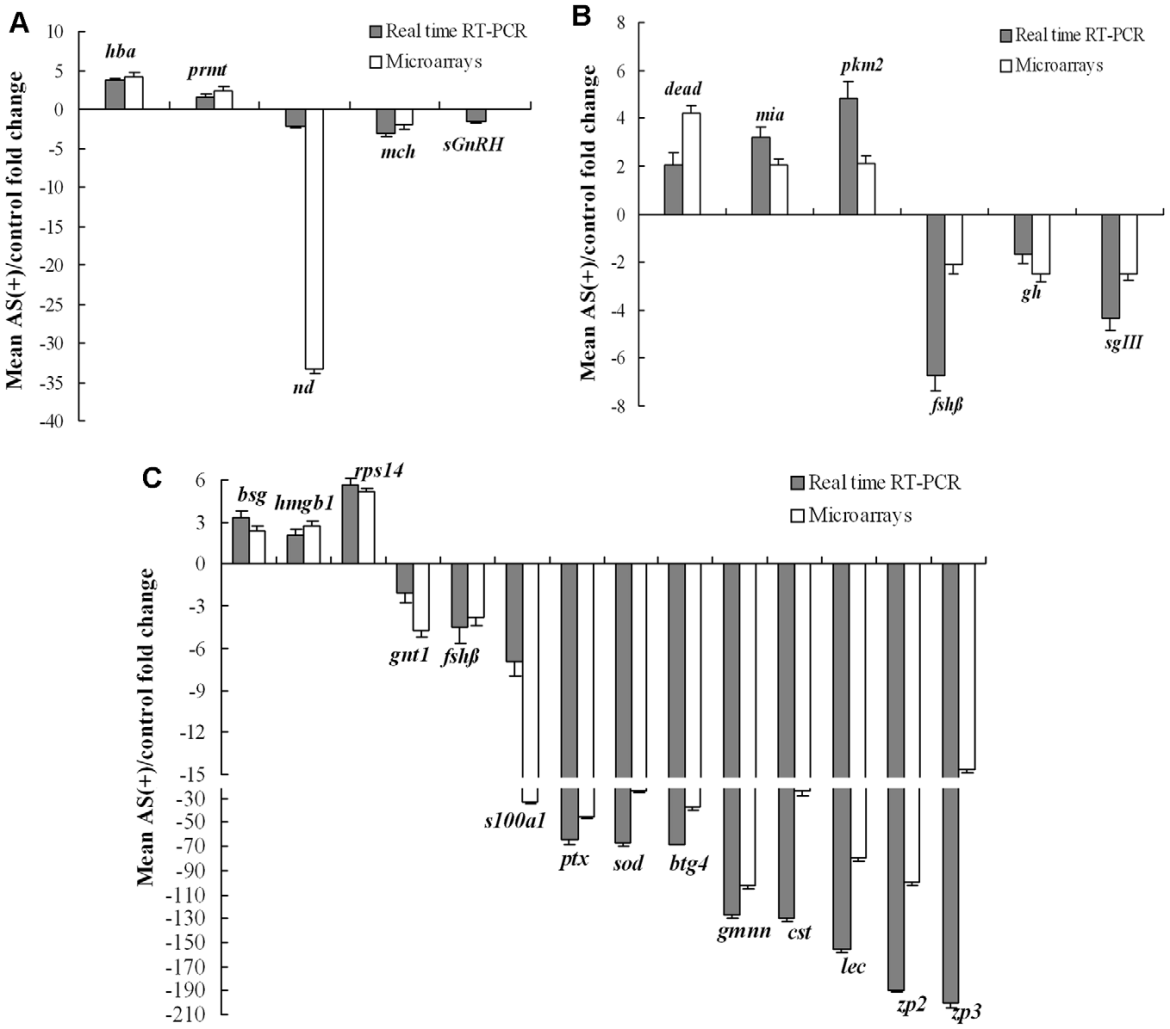
Validation of the differentially expressed genes using real-time quantitative RT-PCR. (A) Hemoglobin subunit alpha (*hbα*), NADH dehydrogenase (*nd*), arginine methyltransferase (*prmt*), melanin-concentrating hormone precursor (*mch*), salmon gonadotropin-releasing hormone (*sGnRH*) in hypothalamus. (B) Growth hormone (*gh*), DEAD box polypeptide (*dead*), similar to melanoma inhibitory activity protein (*mia*), follicle stimulating hormone beta-subunit (*fshβ*), pyruvate kinase M2 (*pkm2*), secretogranin III (*sgIII*) in pituitary. (C) Cystatin precursor (*cst*), C-type lectin (*lec*), B-cell translocation gene 4 (*btg4*), GABA neurotransmitter transporter 1 (*gnt1*), high-mobility group box 1 (*hmgb1*), basigin (*bsg*), ZP2 (*zp2*), follicle stimulating hormone beta-subunit (*fshβ*), 40S ribosomal protein S14 (*rps14*), S100 calcium binding protein A1 (*s100a1*), ZP3 (*zp3*), superoxide dismutase (*sod*), geminin (*gmnn*), pentraxin (*ptx*) in ovary. *β-actin* was amplified with the target gene as a positive control in each well.

In some cases, a much higher-fold change was obtained from real-time RT-PCR than that from microarray results (Figure 5). This might be due to a low concentration of cDNA resulting in a relatively low dynamic range in microarrays [19], [20]. The overall results of real-time RT-PCR agreed with the microarray data (Figure 5). In addition, the level of endogenous sGnRH expression was also detected using real-time RT-PCR of the hypothalamus of AS(+) and control carp. The result showed about 1.5-fold reduction from the former to the latter. These findings support the credibility of the SSH and microarray analysis results.

### Plasma levels of LHβ

Microarray analysis revealed the steady-state expression of various important neuropeptides, hormones, metabolic, oogenesis, and inflammatory factors. Especially in the pituitary, gonadotropin common α subunits and FSHβ subunits were both down-regulated and repeated many times in the SSH library, but the expression of LHβ was not affected. To confirm this result, plasma levels of LHβ were checked by sandwich Enzyme-linked Immunosorbent Assay (ELISA) [21]. The mean plasma LHβ levels of AS(+) and the control group were 0.19±0.025 ng/ml and 0.37±0.048 ng/ml, *P*>0.05. This result showed that LHβ level of the two groups were not significantly different (Table 4). Therefore, these results support the patterns found in our analysis of SSH and microarray data.

**Table 4:** Plasma LHβ level of AS(+) and control groups in 4-year-old female fish.

	Samples	Mean LHβ level(ng/ml)
AS(+) group	n = 6	0.19±0.025
Control group	n = 6	0.37±0.048
*t*-test		*P*>0.05

## Discussion

In teleosts, abnormal migration or mistargeting of GnRH neurons during embryogenesis has been shown by transgenic techniques in Atlantic salmon (*Salmo salar*), rainbow trout, and tilapia (*Oreochromis niloticus*), and the results showed that hypogonadism and infertility could occur [12], [13], [22]. Our laboratory has also generated transgenic common carp expressing sGnRH-antisense RNA, using an improved recombinant vector. As reported earlier, unilateral or undeveloped testes were found in some 14-month-old AS(+) male carp, while no morphological or histological differences were observed in the AS(+) and control females at the same age [14]. However, in the present study, unilateral and undeveloped ovaries were dissected from the remaining 4-year-old AS(+) female carp. One reason for the difference in females was that under natural conditions female carp took two to three years to mature sexually, while male carp could become sexually mature in one year. The gonads of 14-month-old females were incompletely developed for both the AS(+) and the control, and clearly morphological and histological changes could not be observed. Another reason may be that only 5 females were dissected randomly in the previous report and number was too small for us to obtain the carp with undeveloped ovaries.

Since the AS(+) carp with abnormal ovaries selected for SSH and microarray were founders, the antisense sGnRH was expressed as a mosaic. To eliminate the influence of mosaics, tail clip PCR and RT-PCR were performed to verify the functional integration of antisense sGnRH in AS(+) carp used in the present study. Tail clip PCR revealed that the construct was stably integrated in the genome. RT-PCR revealed the antisense sGnRH was expressed in the hypothalamus (Figure S1) and endogenous sGnRH was down-regulated (Figure 5A). The reduced sGnRH in the hypothalamus may influence gene expressions in the pituitary and ovary through the HPG axis. Gene expression surveys of ovary or testis have been carried out in recent years in a number of vertebrates, including coho salmon (*Oncorhynchus kisutch*), European sea bass (*Dicentrarchus labrax*), flatfish (*Heterosomata*), trout, zebrafish and kuruma shrimp (*Marsupenaeus japonicus*) [23]–[28]. Hypothalamic transcriptome analyses were applied in goldfish to study the neuroendocrine regulation. Changes of mRNA levels following various treatments were relatively modest in hypothalamus compared to other tissues [9], [29], [30], since disregulation of hypothalamus eventually leads to complete failure of control mechanisms. In the present study, AS(+) female carp were used as specific variations to check gene expression changes in the tissues of hypothalamus, pituitary and ovary. As a result, differentially expressed candidate genes were isolated specifically correlating to the hypothalamus, pituitary, and ovary subtraction libraries (Tables 1–3). The number of differentially expressed genes was smallest in the hypothalamus while was the largest number was in the ovary, indicating the progressive scaling-up effect of hypothalamic sGnRH antisense. The effect was consistent with an existing theory whereby sGnRH acts as the start signal molecule of the HPG axis pathway [31]. Of interest, most genes identified in the library were neuropeptides, enzymes, growth hormones, and immune molecules, in addition to a major cluster of ribosomal/mitochondrial genes.

In the present study, the neuropeptide melanin-concentrating hormone precursor (*mch*) is down-regulated in both hypothalamus and ovary libraries of AS(+). The protein MCH has been recorded in the brains of organisms ranging from mammals to fish, and functions in several physiological processes, such as food intake, osmoregulation and lactation [32]. With respect to reproduction, studies of female rats have shown that MCH is capable of stimulating the luteinizing hormone-releasing hormone (LHRH) which also known as GnRH from the median eminence and gonadotropin release from the pituitary [33]. In teleosts, Amiya et al. [34] have provided anatomical evidence in barfin flounder (*Verasper moseri*) showing that GnRH immunoreactive fibers were in close contact with MCH cell bodies in the hypothalamus. More recently, *in vitro* experiments on goldfish pituitary cells by Tanaka et al. [35] found that graded doses of MCH could stimulate the secretion of LH while suppressing somatolactin (SL) release via the MCH receptor. Hence our results, together with the published literature, suggest that hypothalamic MCH may play a role as a neuromodulator involved in controlling the release of gonadotropins associated with sGnRH.

FSH and LH, formerly termed GtH I and GtH II, are the two principal gonadotropins in vertebrates, and consist of a common α-subunit and the corresponding hormone-specific β-subunit FSHβ or LHβ [36]. Previous studies have indicated that these subunits play important, but different, roles in the reproductive system. It is believed that FSHβ controls growth stages, including puberty and gametogenesis. Its expression is at high levels during early vitellogenic stages. LHβ controls the maturation phases, including gonadal maturation and spawning. Its expression is lower during the vitellogenic stages and increases toward spawning [37], [38], [39]. In the present study, AS(+) and the control carp were sampled in January when normal gonads were at the previtellogenic or vitellogenic stages. Compared with the control group, common α and FSHβ were observed to be strongly down-regulated in the AS(+) pituitary (Table 2) while LHβ was not detected in the libraries and its plasma level in AS(+) indicated no significant difference (Table 4). The different expression patterns of FSHβ and LHβ observed in our study are consistent with their functional differences in controlling fish reproduction.

Meanwhile, growth hormone (GH), also known as somatotropin in fish, was found to be down-regulated in both pituitary and ovary libraries of the AS(+) carp. In recent years, it has been established that the pituitary growth hormone is involved in the reproductive system of a number of teleost species, in addition to the obligatory role in body growth. The secretion of GH is regulated by hypothalamic neuroendocrine factors, and is capable of stimulating ovarian steroidogenesis [2], [40], [41]. Therefore, a reduction in the expression of GH is not unexpected in transgenic carp, due to the degradation of sGnRH and the incomplete status of ovary development.

Alterations in steroid levels may serve as a stimulus signaling the alteration of teleosts' metabolic processes, because these fluctuations could be translated by neuroendocrine mechanisms into signals that, in turn, alter the pattern of food intake and subsequent energy balance [42], [43]. Our study supported this viewpoint, with a large number of metabolic-related factors being detected, including cytochrome c oxidase subunits I, II, III, NADH dehydrogenase subunits, and ATP synthetase varying in AS(+) tissues, as well as a large number of ribosomal/mitochondrial genes (22.0%). Cytochrome c oxidase is a large transmembrane protein complex, containing three common subunits, I, II and III. As a key enzyme in aerobic metabolism, cytochrome c oxidase catalyzes the transfer of electrons from reduced cytochrome c to molecular oxygen [44]. NADH dehydrogenase, also called complex I, is an enzyme located in the inner mitochondrial membrane that catalyzes the transfer of electrons from NADH to coenzyme Q [45]. The two enzymes are considered to be vital to ATP synthesis in organisms. Recently, Tingaud-Sequeira et al. [25] and Lake et al. [46] independently reported that cytochromes and NADH were up-regulated during the vitellogenesis of flatfish and in the dietary phosphorus of the rainbow trout. These findings suggest the putative mitochondrial function of high energy production. In the present study, the cytochrome c oxidase subunit I was detected as down-regulated in all three tissue libraries. On the other hand, the NADH dehydrogenase subunit I was down-regulated only in the hypothalamus and pituitary libraries (Tables 1–3). These results may be related to the reduced energy expenditure requirement of the dysfunctional ovary of AS(+) carp. However, the NADH dehydrogenase subunit I and ATP synthase, a constituent of the respiratory chain responsible for ATP synthesis, were detected as up-regulated in the ovary library (Table 3). These adaptations may comprise a vital compensatory response to the abnormal conditions of the transgenic ovary.

An abnormal ovary is the greatest variation in the biology of AS(+) females as opposed to the control carp, hence ovarian transcripts involved in the development procession are likely to be affected. Indeed, a set of genes were confirmed as down-regulated in the ovary libraries based on the combined SSH and microarray analyses. These included cathepsin L (*catl*), zona pellucida glycoprotein 2 (*zp2*), zona pellucida glycoprotein 3 (*zp3*), tissue inhibitor of metalloproteinase 4 (TIMP4), and ovulatory protein-2 precursor (Table 3). Cathepsin L is a member of the papain-like family of cysteine proteinases. Robker et al. [47] has reported in mouse that Cathepsin L could be induced by FSH in the granulosa cells of growing follicles, while high levels of *catl* mRNA could be also induced by luteinizing hormone in a progesterone receptor-dependent fashion in pre-ovulatory follicles. In zebrafish, *catl* was also reported to be associated with ovarian follicle degeneration [48]. The zona pellucida (ZP) is an extracellular glycoprotein matrix which surrounds all mammalian oocytes. Recent teleost researches have shown the synthesis of ZP transcripts and proteins involved in yolk incorporation (vitellogenesis) and processing of primary growth [23], [27]. Tissue inhibitors of metalloproteinases (TIMPs), which comprise a family of four proteases TIMP1, TIMP2, TIMP3 and TIMP4, are inhibitors of the matrix metalloproteinases (MMPs), a group of peptidases that are involved in the degradation of the extracellular matrix. Studies in mammals have indicated that MMPs/TIMPs might regulate normal follicular development and atresia to achieve the appropriate number of ovulatory follicles [49]. As to the ovulatory protein-2 precursor, previous studies in trout have described its dramatic up-regulation at the time of ovulation, and suggested a role in protecting ovulated eggs from bacterial infection, in addition to the concurrent function of ovulation [50]. Therefore, according to the published literature, the down-regulation of these genes in this study indicate that they are involved in the incomplete development process of the AS(+) ovary, related to the ovary adhesion tissues, vitellogenesis, oocyte maturation and ovulation processes, respectively.

In teleosts, several elegant studies of flatfish, rainbow trout, and salmon have indicated that inflammatory factors may be involved in the process of ovary development. These factors include serine protease 23 (*sp23*), disintegrin and metalloproteinase domain-containing protein 22 (*adam22*), Chemokine (C-X-C motif) ligand 14 (*cxcl14*), angiotensin I converting enzyme 2 (*ace2*), and leukocyte cell-derived chemotaxin 2 (*lect2*) [25], [27], [51]. The present study has provided further evidence validating the involvement of inflammatory factors in the process of ovary development, whereby several immune genes (C-type lectin, pentraxin, and metallothionein-I, -II) were detected to be strongly down-regulated in the AS(+) ovary (Table 3). It has been reported that the C-type lectins represent a large family, containing a common carbohydrate recognition domain that interacts with glycoproteins in a Ca^2+^-dependent manner [52], [53]. This family has been found throughout the animal kingdom, and is involved in many immune-system functions, such as innate immunity, or the inflammation and immunity response to tumor and virally infected cells [54]. The pentraxins are another type of immune gene, and are a family of proteins characterized by a pentagonal discoid arrangement of five non-covalently bound subunits similar to that of legume lectins [53]. Pentraxins have been found to be important for innate defense, particularly the acute phase response, in both mammals and fish [54], [55]. The metallothioneins (MTs) have been described in a wide range of taxonomic groups, and are involved in heavy metal detoxification and homeostasis [56]. In our study these immune factors were obtained from the undeveloped ovary of the AS(+) carp, which based on the existing literature suggests that these genes may be involved in the process of teleost ovary development.

In contrast to the genes mentioned above, basigin was detected to be up-regulated in the AS(+) ovary. This gene is a member of the immunoglobulin superfamily that is also known as CD147, BSG, and EMMPRIN. The fundamental role of this gene is in intercellular recognition, including a range of immunologic phenomena, differentiation, and development. It has also been suggested that basigin may regulate mouse spermatogenesis, follicular development, and oocyte maturation [57]. In teleosts, basigin has been expressed in follicle/interstitial cells that are associated with previtellogenic growth in coho salmon [51]. Hence, our findings support those of the previous studies, whereby the increased expression of this gene may contribute to oocyte development in the abnormal ovary.

The data relating to the differentially expressed genes identified in this study with respect to the HPG signaling pathway are far more extensive than those mentioned above. Of note, the presented genes that have yet to be named with unknown functions, or span multiple functional categories have not been considered in this discussion, including tissue remodeling, ion transportation, apoptosis, cell-cycle progression, and growth. These have not been considered in this discussion and are a valuable resource for further investigation.

In conclusion, this study provided novel information on transgenic common carp with abnormal ovaries as a result of sGnRH-antisense RNA expression. This study also provided comprehensive data with respect to changes in gene expression throughout the HPG signaling pathway, as a result of employing combined SSH and microarrays. Moreover, significant gene families that were represented in the SSH libraries were highlighted, including neuropeptides, gonadotropins, metabolic, oogenesis and inflammatory factors. Finally, a list of 87 candidate novel genes was successfully generated, requiring further analyses to validate their uniqueness and roles. Therefore, this study not only indicated the progressive scaling-up effect of hypothalamic sGnRH antisense acting on the pituitary and ovary, but also provided new insights with respect to the understanding of the molecular mechanisms and regulative pathways in the reproductive system of teleosts.

## Materials and Methods

### Ethics Statement

The animals are provided with the best possible care and treatment and are under the care of a specialized technician. Also, all animals are cared for and handled with respect. All procedures were conducted in accordance with the Guiding Principles for the Care and Use of Laboratory Animals and were approved by Institute of Hydrobiology, Chinese Academy of Sciences (Approval ID: keshuizhuan08529).

### Screening of female transgenic common carp

The transgenic common carp with an antisense sGnRH construct were as described in our previous report [15]. In brief, the antisense sGnRH construct which contained a carp *β-actin* gene promoter, a 328-bp antisense DNA fragment and the 3′ flanking sequence of grass carp growth hormone gene was injected into freshly fertilized eggs from wild parent carp in April 2003. The same age non-transgenic siblings of the transgenic carp were raised as controls. Transgenic and non-transgenic carp were raised communally in secure tanks in Wuhan Duofu Scientific & Technological Farm Co., Ltd. After eight months, the transgenic carp were screened for the presence of the transgene using PCR. A total of 342 putative transgenic carp were assayed, of which 102 were obtained with the antisense construct. Carp retaining the transgene were named AS(+).

In every breeding season (usually April) of the next four years, the AS(+) carp were pressed lightly on the abdomen and sperm could be extracted from the fertile males. Eggs could be extracted from those with normal ovaries with a tool made specially for female carp (copper, 10 cm long, with a 1.5 cm long and 0.3 cm wide groove in the anterior part). The remaining carp without sperm and eggs were tagged by coded wire tags (Northwest Marine Technology, Inc.) and the statistical data was put on record. In Jan 2008, the tagged AS(+) carp which could not produce mature gametes were dissected. Twelve AS(+) carp with abnormal ovaries and 10 four -year-old non-transgenic female siblings were selected for our analysis. Their blood was collected from the caudal vasculature and the tissues of hypothalamus, pituitary and gonads were dissected and frozen in liquid nitrogen immediately before storing at −80°C. At the same time, parts of the gonads were fixed in Bouin's solution for histological examination. Fixed gonads were dehydrated and embedded in paraffin, and 5 µm sections were cut and stained with Regaud Haematoxylin-Orange G-Aniline blue.

Tail clip PCR and RT-PCR were used to evaluate the expression of sGnRH antisense RNA in the genome and hypothalamus of 12 female AS(+) carp. Genomic DNA was extracted from the caudal fin using DNeasy Blood & Tissue Kit (Qiagen), and total RNA was extracted from the hypothalamus using Trizol (Invitrogen) reagent according to the manufacturer's instructions. Antisense sGnRH were amplified by the primers P1 (5′-CCATGG-CGTATCGATGTCGAC-3′) and P2 (5′-CATGGCTTTGCCAGCATTGG-3′). The PCR program was as follows: 1 cycle of 90°C for 1 min, 35 cycles of 90°C for 20 s, 55°C for 30 s, 72°C for 50 s; 1 cycle of 72°C for 5 min. *β-actin* was amplified as a positive control and the sequences of *β-actin* primers were ACT-F, 5-CACTGTGCCCATCTACGAG-3 and ACT-R, 5-CTGCATCCTGTCAGCAATGC-3.

### Total RNA extraction and cDNA synthesis

Tissue samples of the hypothalamus, pituitary, and ovary were collected from three AS(+) and three control carp, respectively. Total RNAs were extracted using the TRIzol reagent (Invitrogen) and purified using PolyA Tract mRNA Isolation System (Promega) following the recommended guidelines. RNA quality and purity was measured using a spectrophotometer (Eppendorf Biometer) and electrophoresis on 1% agarose gels. RNA from the hypothalamus of three AS(+) carp were pooled together and RNA from three control carp were similarly pooled together, as were RNA from the pituitary and ovary. SMART cDNA was synthesized from 50 ng of total RNA using the SMART cDNA Library Construction Kit following a commercial protocol (Clontech) as described previously [58]. Subsequently, double-strand cDNAs were produced through PCR amplification and purified by phenol:chloroform:isoamyl alcohol (25∶24∶1) extraction.

### Suppressive subtractive hybridization

To identify the genes involved in the HPG axis pathway that were differentially expressed between transgenic and control carp, we used SSH as a technique to create six sublibraries of the three levels of hierarchy from hypothalamus to pituitary, and to ovary, respectively. The purified double-stranded cDNA obtained from AS(+) was used as the “tester”, while the cDNA obtained from control carp served as the “driver” (forward SSH library). Conversely, cDNAs from AS(+) and control carp were also used as driver and tester samples, respectively (reverse SSH library). Construction of the forward and reverse libraries was performed using a PCR-select cDNA subtraction kit (Clontech) according to the SSH procedure. Briefly, cDNA from each of the tester and driver populations were digested with *Rsa* I to produce shorter blunt-ended fragments. The digested tester cDNAs were then subdivided into two populations, each of which was ligated with a different adaptor, provided in the cDNA subtraction kit. PCR was performed to evaluate the efficiency of ligation using primers specific to *β-actin* and to the adaptor sequences. Following ligation, two rounds of hybridization and PCR amplification were performed. In the first hybridization step, the driver was added in excess to each tester, denatured, and allowed to anneal. In the second hybridization step, the two products from the first hybridization were mixed together, in addition to freshly denatured cDNA driver. Subsequently, the populations of normalized and subtracted single-strand target cDNA samples annealed with each other, forming double-stranded hybrids with different adaptor sequences at their 5′ ends. Finally, the subtracted molecules were specifically amplified using adaptor-specific primer pairs of ‘nested PCR’.

### Construction of subtracted cDNA libraries

PCR-amplified cDNAs produced by SSH were ligated into the pMD18-T plasmid vector (TaKaRa) and transformed into competent *Escherichia coli* (strain DH5α) by electroporation (Pulse Controller, BioRad, USA). The transformed bacteria were plated onto solid Luria Bertani medium containing ampicillin, X-gal and IPTG, and incubated overnight at 37°C. Of note, pMD18-T plasmid contains LacZ reporter which allows blue-white screening. About 20,000 recombinant white clones were randomly selected and amplified in a 100 µl PCR system using nested primer 1 and 2R (Clontech) for positive detection. Aliquots (1 µl) of the PCR products were analyzed in 1% agarose gel to verify the quality and quantity. Then, the single-insert clones with amplified fragments 200–1,000 bp were selected for the construction of cDNA chips.

### Microarray construction

Numbers of cDNA microarray chips containing cDNA spots representing 15,998 SSH clones, of which 4,996 clones were from the hypothalamus libraries, 4,992 from the pituitary libraries, and 6,000 from the ovary counterparts, were constructed. Briefly, the PCR products of single-insert clones were purified by the chilled ethanol precipitation method and redissolved in 15 µl of 50% dimethyl sulphoxide (DMSO), and finally spotted onto amino-silaned glass slides with a SmartArrayer™ microarrayer (CapitalBio Corp., Beijing, China). Each clone was printed in duplicate. The slides were baked for 1 h at 80°C and then stored at room temperature until use.

On each microarray chip, eight sequences derived from intergenic regions in yeast genome, showing no significant homology to common carp in GenBank, were spotted as external controls. And two housekeeping genes of common carp (*β-actin* and Glyceraldehye-3-Phosphate Dehydrogenase [GAPDH]) were used as internal controls. Additionally, 50% DMSO was used as a negative contol for subtracting the background, and Hex was positive control for nucleic acid fixation.

### Preparation of fluorescent probes and hybridization of microarrays

Total RNA samples of the hypothalamus, pituitary and ovary, obtained from three additional AS(+) and control carp, were extracted using the standard TRIzol RNA isolation protocol and purified as mentioned above. Briefly, 5 µg of each isolated RNA was respectively reverse transcribed with an oligo (dT) 8–12 (Promega), Cy3/Cy5 CTP, and Superscript II reverse transcriptase (Invitrogen Life Technologies, Shanghai, P.R. China). In order to prepare the cDNA probes, Cy3 and Cy5 dyes (Amersham, Piscataway, NJ) were used to label the cDNA isolated from AS(+) and control tissue samples. All probes were purified and later hybridized with the spotted array at 42°C for 16 h. The slides were then washed once with 2× SSC-0.1% SDS at 42°C for 10 min, and four times with 0.1× SSC at room temperature for 1 min. Finally, the slides were washed with distilled water, ethanol, and then dried. Each hybridization step was performed twice for replicate dye-swaps.

### Microarray data analysis

Arrays were scanned with a confocal laser scanner, LuxScan™ 10K (CapitalBio Corp.), and the resulting images were analyzed with SpotData Pro 2.0 software (CapitalBio Corp.). Spots with fewer than 50% of the signal pixels exceeding the local background value for both channels (Cy3 and Cy5) plus two standard deviations of the local background were removed. This step further ensured that spots with characteristic doughnut shapes, often encountered on microarrays, would not be part of the subsequent analysis. A spatial and intensity-dependent (LOWESS) normalization method was employed to normalize the ratio values [59]. Normalized ratio data were then log transformed. cDNA spots with less than four out of total six data points in each replicated hybridization were removed. Differentially expressed genes were identified using *t* test and multiple test corrections were performed using False Discovery Rate (FDR). Genes with FDR <0.01 and a fold change greater than or equal to two were identified as differentially expressed genes.

### DNA sequence analysis and gene ontology analysis

The identified differentially expressed clones were sequenced using the M13± primer pairs (Invitrogen Life Technologies). Sequences were analyzed with the Basic Local Alignment Search Tool (BLAST) in NCBI for homology. cDNAs with *E* values ≤1e−5 were designated as having significant homology, and the higher score affirmed the corresponding gene. Functional categories of the identified genes were assigned based on the Gene Ontology annotations (http://www.geneontology.org/).

### Validation of differentially expressed genes by real-time RT-PCR

The differentially expressed genes were further validated by real-time quantitative RT-PCR that were run on an ABI 7000 fluorescent sequence detection system (Perkin-Elmer, Foster City, CA), with SYBR green-based detection (ABI) using gene-specific primer pairs. In practice, 25 genes were chosen for the real-time RT-PCR analysis, five of which were tested in hypothalamus, six in pituitary, 14 in ovary. *β-actin* was used as the control housekeeping gene since it has been found not to vary across organs in carp [60] and is not affected by GnRH [61], [62]. The primers of these genes are listed in Table S5. Total RNA came from three additional AS(+) and control carp specimens. Reactions were performed using the following conditions: an initial incubation at 95°C for 5 min, followed by 40 cycles at 95°C for 15 s, 55°C for 15 s and 72°C for 45 s. Output data generated by the instrument onboard software were transferred to a custom-designed Microsoft (Redmond, WA) Excel spreadsheet for analysis. The differential mRNA expression of each sample was calculated as previously described by the comparative Ct method with the formula 2^(-Delta Delta C(T))^ method [63], [64]. The experiments were conducted independently for each of the hypothalamus, pituitary and ovary from the AS(+) carp with unilateral gonad and their control siblings. Each reaction was performed in triplicate, with the means being evaluated using the Student *t*-test (P<0.01).

### Sandwich ELISA to detect plasma LHβ levels

To detect levels of LHβ, blood samples from an additional six AS(+) and six control carp were collected from the caudal vasculature, kept on ice, and later centrifuged to obtain plasma samples which were then examined by the sandwich ELISA system developed in our previous report [21]. In brief, Anti-LHβ mAb (5 µg/mL) in coating buffer (0.05 M carbonate/bicarbonate buffer, pH 9.5) was coated at 100 µL/well on a 96-well ELISA plate (Nunc, Denmark), incubated overnight at 4°C and washed 3 times with PBS containing Tween-20 (PBS/Tween). Plates were then blocked with a 0.1% BSA-PBS. Standard LHβ serial dilutions with PBS containing BSA and Tween-20 or plasma samples of carp were added to the wells and incubated. After extensive washing with PBS/Tween, wells were incubated with 100 µL/well of anti-LHβ mAbs conjugated with HRP. Wells were washed once again for 3 times and 100 µL of 2,2′-azobis-3-ethylbenzthiazoline-6-sulfonic (ABTS, Vector) was added to each well. Plates were read at 410 nm on a microplate reader. Comparison of the mean LHβ concentration in the plasma of AS(+) and control groups was performed using the Student's *t*-test.

## Supporting Information

Figure S1 **Expression of antisense sGnRH in genome and hypothalamus of the selected 12 AS(+) female carp.** 1–12 was the number of the AS(+) carp and M was DL2000 DNA marker. *β-actin* was amplified as a positive control. The length of PCR products for antisense sGnRH and *β-actin* was 328 bp and 460 bp respectively. The first band of molecular weight marker (M) was 500 bp, and the second was 250 bp.
(DOC)

Figure S2 **Gross morphology of four-year-old AS(+) and normal fish.** (A) abnormally developed ovary morphology of AS(+) carp. (B) normally developed ovary morphology of normal carp. The portions of the ovary tissue are outlined with white elliptic boxes, respectively.
(DOC)

Figure S3 **An example of part of one microarray screening for SSH cDNA libraries.** cDNA microarrays were hybridized separately using fluorescent (Cy3 and Cy5 dyes) labeled probes prepared from AS(+) and control carp mRNA of the hypothalamus (A), pituitary (B), and ovary (C). Red spots indicate the relative overexpression in AS(+), and green spots indicate the relative overexpression in control carp. Yellow spots indicates equal expression in both carp types.
(DOC)

Table S1 **Reproductive status of transgenic common carp.**
(DOC)

Table S2 **A full list of differentially expressed genes in the hypothalamus subtracted library of AS(+) carp. (FDR <0.01 and fold change ≥2).**
(DOC)

Table S3 **A full list of differentially expressed genes in the pituitary subtracted library of AS(+) carp. (FDR <0.01 and fold change ≥2).**
(DOC)

Table S4 **A full list of differentially expressed genes in the ovary subtracted library of AS(+) carp. (FDR <0.01 and fold change ≥2).**
(DOC)

Table S5 **Primers used to detect 25 chosen genes in real-time quantitative RT-PCR.**
(DOC)
