# Supplementary material for: Defining Global Gene Expression Changes of the Hypothalamic-Pituitary-Gonadal Axis in Female sGnRH-Antisense Transgenic Common Carp (Cyprinus carpio)
Source: PLoS One. 2011 Jun 10;6(6):e21057. doi: 10.1371/journal.pone.0021057 (PMC3112210; doi:10.1371/journal.pone.0021057)
Supplement: Figure S1 — Expression of antisense sGnRH in genome and hypothalamus of the selected 12 AS(+) female carp. 1–12 was the number of the AS(+) carp and M was DL2000 DNA marker. β-actin was amplified as a positive control. The length of PCR products for antisense sGnRH and β-actin was 328 bp and 460 bp respectively. The first band of molecular weight marker (M) was 500 bp, and the second was 250 bp. (DOC) [file pone.0021057.s001.doc]

**Genome**

**Hypothalamus**

***β-actin***

**M 1 2 3 4 5 6 7 8 9 10 11 12**

***Antisense sGnRH***


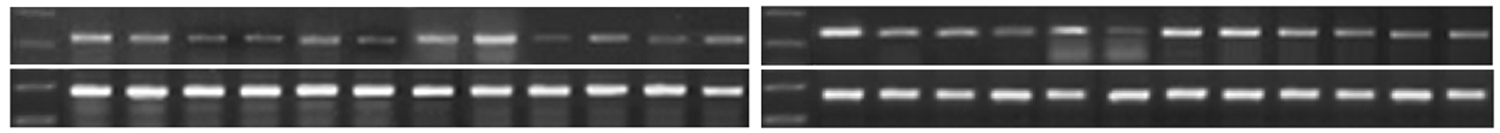


**M 1 2 3 4 5 6 7 8 9 10 11 12**

**Figure S1: Expression of antisense sGnRH in genome and hypothalamus of the selected 12 AS(+) female carp.** 1~12 was the number of the AS(+) carp and M was DL2000 DNA marker. β-actin was amplified as a positive control. The length of PCR products for antisense sGnRH and β-actin was 328 bp and 460 bp respectively. The first band of molecular weight marker (M) was 500 bp, and the second was 250 bp.
