# Supplementary material for: Defining Global Gene Expression Changes of the Hypothalamic-Pituitary-Gonadal Axis in Female sGnRH-Antisense Transgenic Common Carp (Cyprinus carpio)
Source: PLoS One. 2011 Jun 10;6(6):e21057. doi: 10.1371/journal.pone.0021057 (PMC3112210; doi:10.1371/journal.pone.0021057)
Supplement: Figure S2 — Gross morphology of four-year-old AS(+) and normal fish. (A) abnormally developed ovary morphology of AS(+) carp. (B) normally developed ovary morphology of normal carp. The portions of the ovary tissue are outlined with white elliptic boxes, respectively. (DOC) [file pone.0021057.s002.doc]

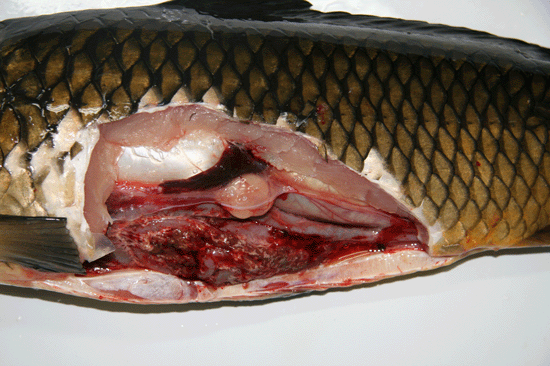

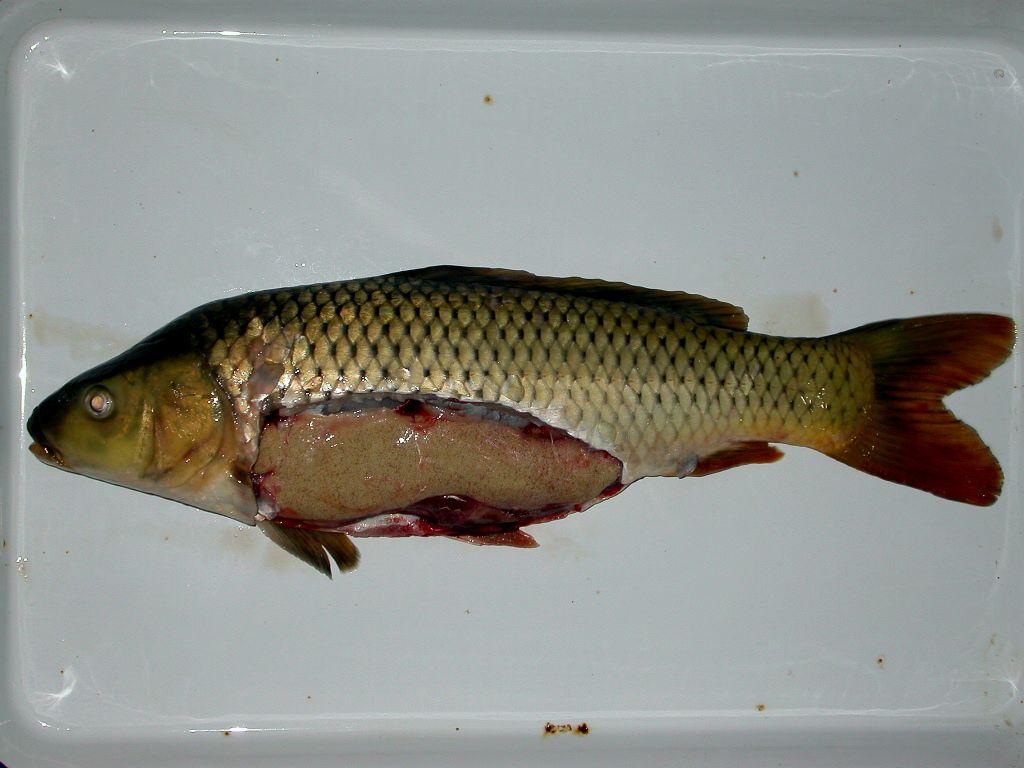


**A**

**B**

**Figure S2: Gross morphology of four-year-old AS(+) and normal fish.** (A) abnormally developed ovary morphology of AS(+) carp. (B) normally developed ovary morphology of normal carp. The portions of the ovary tissue are outlined with white elliptic boxes, respectively.
