# Supplementary material for: Defining Global Gene Expression Changes of the Hypothalamic-Pituitary-Gonadal Axis in Female sGnRH-Antisense Transgenic Common Carp (Cyprinus carpio)
Source: PLoS One. 2011 Jun 10;6(6):e21057. doi: 10.1371/journal.pone.0021057 (PMC3112210; doi:10.1371/journal.pone.0021057)
Supplement: Figure S3 — An example of part of one microarray screening for SSH cDNA libraries. cDNA microarrays were hybridized separately using fluorescent (Cy3 and Cy5 dyes) labeled probes prepared from AS(+) and control carp mRNA of the hypothalamus (A), pituitary (B), and ovary (C). Red spots indicate the relative overexpression in AS(+), and green spots indicate the relative overexpression in control carp. Yellow spots indicates equal expression in both carp types. (DOC) [file pone.0021057.s003.doc]

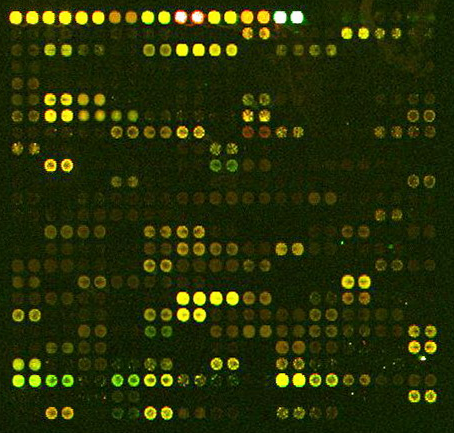

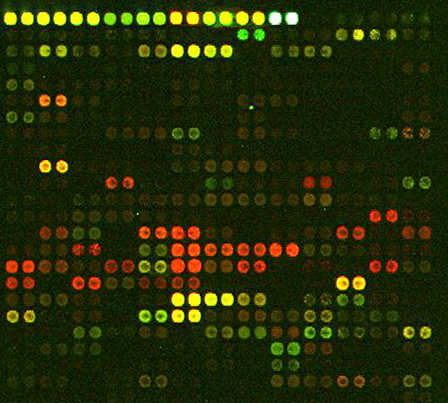

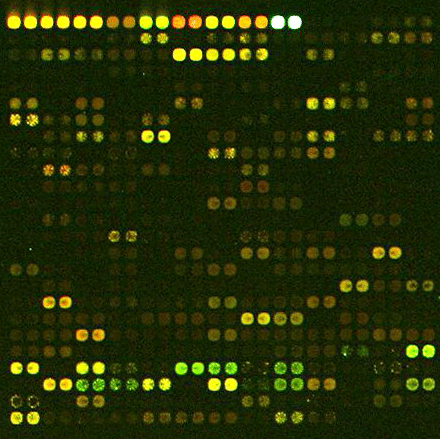


**A**

**C**

**B**

**Figure S3: An example of part of one microarray screening for SSH cDNA libraries.** cDNA microarrays were hybridized separately using fluorescent (Cy3 and Cy5 dyes) labeled probes prepared from AS(+) and control carp mRNA of the hypothalamus (A), pituitary (B), and ovary (C). Red spots indicate the relative overexpression in AS(+), and green spots indicate the relative overexpression in control carp. Yellow spots indicates equal expression in both carp types.
