# Supplementary material for: Defining Global Gene Expression Changes of the Hypothalamic-Pituitary-Gonadal Axis in Female sGnRH-Antisense Transgenic Common Carp (Cyprinus carpio)
Source: PLoS One. 2011 Jun 10;6(6):e21057. doi: 10.1371/journal.pone.0021057 (PMC3112210; doi:10.1371/journal.pone.0021057)
Supplement: Table S1 — Reproductive status of transgenic common carp. (DOC) [file pone.0021057.s004.doc]

**Table S1.** Reproductive status of transgenic common carp.

|  | Total | With normal gonadal development | | With abnormal gonadal development | | Without gonad | Mean LH level(ng/ml) |
| --- | --- | --- | --- | --- | --- | --- | --- |
|  |  | Male | Female | Male | Female |  |  |
| Number | 102 | 33 | 31 | 14 | 12 | 12 | 5.63±0.44 ng/ml (n=65)  Tested  in April, 2004 |
| Percentage | 100% | 32.4% | 30.4% | 13.7% | 11.8% | 11.8% |  |
