# Supplementary material for: Defining Global Gene Expression Changes of the Hypothalamic-Pituitary-Gonadal Axis in Female sGnRH-Antisense Transgenic Common Carp (Cyprinus carpio)
Source: PLoS One. 2011 Jun 10;6(6):e21057. doi: 10.1371/journal.pone.0021057 (PMC3112210; doi:10.1371/journal.pone.0021057)
Supplement: Table S2 — A full list of differentially expressed genes in the hypothalamus subtracted library of AS(+) carp. (FDR <0.01 and fold change ≥2). (DOC) [file pone.0021057.s005.doc]

| Clone no. | Protein_id | *E* value | Defintion | Clone redundancy in SSH library | Microarray fold change AS(+)/control |
| --- | --- | --- | --- | --- | --- |
| 2f2 | P02016 | 3.00E-75 | Hemoglobin subunit alpha (Alpha-globin) | 2 | 4.14 |
| 73e2 | NP_956944.1 | 2.00E-89 | arginine methyltransferase 1 [Danio rerio] | 2 | 2.27 |
| 6b10 | NP_998170.1 | 2.00E-38 | hypothetical protein LOC406278 [Danio rerio] | 1 | 2.06 |
| 7d8 | XP_690672.2 | 5.00E-22 | hypothetical protein [Danio rerio] | 1 | 2.04 |
| 7e02 | AF249875.1 | 4.00E-81 | Cyprinus carpio metallothionein II mRNA | 1 | 0.50 |
| 6h10 | P56943 | 3.00E-21 | Pro-MCH 1 precursor | 3 | 0.49 |
| 11h1 | AJ293391.1 | 2.00E-16 | Homo sapiens mRNA differentially expressed in malignant melanoma | 8 | 0.46 |
| 12e10 | BAE97651.1 | 2.00E-47 | cytochrome oxidase subunit 1 [Cyprinus carpio] | 1 | 0.39 |
| 7d02 | AB308069.1 | 2.00E-07 | NADH dehydrogenase | 1 | 0.30 |

**Table S2.** A full list of differentially expressed genes in the hypothalamus subtracted library of AS(+) carp. (FDR <0.01 and fold change ≥ 2)
