# Supplementary material for: Defining Global Gene Expression Changes of the Hypothalamic-Pituitary-Gonadal Axis in Female sGnRH-Antisense Transgenic Common Carp (Cyprinus carpio)
Source: PLoS One. 2011 Jun 10;6(6):e21057. doi: 10.1371/journal.pone.0021057 (PMC3112210; doi:10.1371/journal.pone.0021057)
Supplement: Table S3 — A full list of differentially expressed genes in the pituitary subtracted library of AS(+) carp. (FDR <0.01 and fold change ≥2). (DOC) [file pone.0021057.s006.doc]

**Table S3.** A full list of differentially expressed genes in the pituitary subtracted library of AS(+) carp. (FDR <0.01 and fold change ≥ 2)

| Clone no. | Protein_id | *E* value | Defintion | Clone redundancy in SSH library | Microarray fold change AS(+)/control |
| --- | --- | --- | --- | --- | --- |
| 8c11 | XP_687635.1 | 2.00E-16 | hypothetical protein [Danio rerio] | 1 | 7.73 |
| 2f2 | P02016 | 3.00E-75 | Hemoglobin subunit alpha (Hemoglobin alpha chain) (Alpha-globin) | 1 | 4.42 |
| 58f8 | NP_998142.1 | 3.00E-72 | DEAD (Asp-Glu-Ala-Asp) box polypeptide 39 [Danio rerio] | 1 | 4.23 |
| 48g8 | NP_001013513.1 | 1.00E-37 | hypothetical protein LOC541368 [Danio rerio] | 2 | 3.39 |
| 9b11 | XP_001342273.1 | 3.00E-55 | hypothetical protein [Danio rerio] | 1 | 3.11 |
| 7f05 | NP_956317.1 | 2.00E-46 | hypothetical protein LOC336637 [Danio rerio] | 1 | 2.98 |
| 6h12 | CR933528.8 | 2.00E-51 | Zebrafish DNA sequence from clone CH211-144A23 in linkage group 5 | 6 | 2.82 |
| 12c5 | XP_001895031.1 | 2.00E-17 | hypothetical protein Bm1_17870 [Brugia malayi] | 1 | 2.73 |
| 9b10 | NM_200650.1 | 5.00E-35 | Danio rerio protein arginine methyltransferase 1 (prmt1) complete cds | 1 | 2.37 |
| 7b08 | CAK11468.1 | 3.00E-31 | succinate dehydrogenase complex, subunit A, flavoprotein (Fp) [Danio rerio] | 2 | 2.17 |
| 7d06 | AAH67143.1 | 9.00E-30 | Pkm2 protein [Danio rerio] | 1 | 2.13 |
| 9b08 | XP_001336607.1 | 4.00E-26 | similar to melanoma inhibitory activity protein [Danio rerio] | 1 | 2.04 |
| 9c04 | CU104710.8 | 1.00E-35 | Zebrafish DNA sequence from clone CH73-252G14 in linkage group 2 | 1 | 0.50 |
| 7c02 | CT025651.9 | 2.00E-07 | Zebrafish DNA sequence from clone CH211-248K15 in linkage group 4 | 2 | 0.50 |
| 9c12 | Q9YGK5 | 5.00E-48 | Pro-opiomelanocortin (POMC)[Cyprinus carpio] | 2 | 0.49 |
| 7d09 | BAA23757.1 | 2.00E-82 | brain aromatase [Carassius auratus] | 1 | 0.48 |
| 35b1 | X59888.1 | e-141 | C.carpio gene for gonadotropin beta subunit 1(FSHβ) | 124 | 0.47 |
| 43b7 | XM_699646.2 | 1.00E-34 | Danio rerio hypothetical LOC558130 (LOC558130), mRNA | 1 | 0.46 |
| 12e10 | BAE97651.1 | 2.00E-47 | cytochrome oxidase subunit I [Cyprinus carpio] | 2 | 0.46 |
| 7h06 | P01221 | 7.00E-67 | Glycoprotein hormones alpha chain 1 precursor [Cyprinus carpio] | 13 | 0.45 |
| 47f2 | NM_200569.1 | 2.00E-44 | Danio rerio zgc:65831 (zgc:65831) | 1 | 0.44 |
| 7c05 | XP_512771.2 | 2.00E-76 | PREDICTED:similar to calmodulin [Pan troglodytes] | 1 | 0.43 |
| 11g8 | ABX72174.1 | 2.00E-43 | cytochrome oxidase subunit II [Cyprinus carpio] | 1 | 0.42 |
| 8a09 | AAP38173.1 | 1.00E-68 | cytochrome c oxidase subunit III [Carassius auratus] | 3 | 0.42 |
| 11h1 | AJ293391.1 | 2.00E-16 | Homo sapiens mRNA differentially expressed in malignant melanoma | 3 | 0.41 |
| 48a8 | NP_957051.1 | 6.00E-32 | secretogranin III [Danio rerio] | 1 | 0.40 |
| 7f06 | ABY71031.1 | 1.00E-18 | growth hormone [Carassius auratus gibelio] | 2 | 0.40 |
| 7d02 | AB308069.1 | 2.00E-07 | putative NADH dehydrogenase | 2 | 0.21 |
