# Supplementary material for: Defining Global Gene Expression Changes of the Hypothalamic-Pituitary-Gonadal Axis in Female sGnRH-Antisense Transgenic Common Carp (Cyprinus carpio)
Source: PLoS One. 2011 Jun 10;6(6):e21057. doi: 10.1371/journal.pone.0021057 (PMC3112210; doi:10.1371/journal.pone.0021057)
Supplement: Table S4 — A full list of differentially expressed genes in the ovary subtracted library of AS(+) carp. (FDR <0.01 and fold change ≥2). (DOC) [file pone.0021057.s007.doc]

**Table S4. A full list of differentially expressed genes in the ovary subtracted library of AS(+) carp. (FDR <0.01 and fold change ≥ 2)**

| Clone no. | | Protein_id | *E* value | Defintion | Clone redundancy in SSH library | Microarray fold change AS(+)/control |
| --- | --- | --- | --- | --- | --- | --- |
| 15a9 | | NP_001032317.1 | 1.00E-40 | hypothetical protein LOC568735 [Danio rerio] reverse transcriptase | 3 | 23.75 |
| 7d01 | | DQ251457.1 | 5.00E-05 | Siniperca chuatsi transposase mRNA, partial cds | 1 | 21.03 |
| 2f2 | | P02016 | 3.00E-75 | Hemoglobin subunit alpha (Hemoglobin alpha chain) (Alpha-globin) | 2 | 19.96 |
| 6c10 | | XP_001337418.1 | 4.00E-36 | similar to Secretory granule proteoglycan core protein precursor | 1 | 19.34 |
| 15a10 | | NA |  |  | 1 | 15.84 |
| 7b12 | | DQ378978.1 | 5.00E-44 | Carassius auratus gibelio clone CX-J1-20 microsatellite sequence | 1 | 14.07 |
| 15b1 | | XP_689124.2 | 4.00E-11 | hypothetical protein [Danio rerio] | 1 | 12.08 |
| 7c04 | | AB003335.1 | 0 | Carassius auratus mRNA for Y box protein 1, complete cds | 1 | 7.43 |
| 9d12 | | XM_699646.2 | 2.00E-34 | Danio rerio hypothetical LOC558130 (LOC558130), mRNA | 3 | 7.19 |
| 8d11 | | CT027837.9 | 2.00E-44 | Zebrafish DNA sequence from clone DKEY-180C19 in linkage group 19 | 1 | 7.06 |
| 9d05 | | EDL17115.1 | 2.00E-69 | ribosomal protein S15a, isoform CRA_b [Mus musculus] | 3 | 6.88 |
| 15f3 | | CT028615.1 | 6.00E-07 | Poplar cDNA sequences | 2 | 6.59 |
| 7a05 | | NA |  |  | 1 | 6.40 |
| 7c09 | | BX571693.1 | 4.00E-42 | Carp DNA sequence from clone carpf-GC2H, complete sequence | 3 | 6.21 |
| 7d04 | | NP_001018395.1 | 3.00E-60 | hypothetical protein LOC553580 [Danio rerio] | 2 | 6.00 |
| 7c07 | | XP_001338227.1 | 4.00E-77 | similar to transposase (putative) [Danio rerio] | 2 | 5.57 |
| 7f01 | | AAH98591.1 | 2.00E-55 | Ribosomal protein L14 [Danio rerio] | 1 | 5.26 |
| 2f10 | | ABJ98607.1 | 2.00E-73 | 40S ribosomal protein S14 [Scophthalmus maximus] | 3 | 5.15 |
| 5d10 | | NP_957139.1 | 5.00E-67 | hypothetical protein LOC393818 [Danio rerio] | 1 | 5.10 |
| 43c7 | | NP_997908.1 | 3.00E-26 | ribosomal protein, large P2 [Danio rerio] | 1 | 4.94 |
| 7e04 | | CAP09075.1 | 2.00E-30 | minos transposase [Drosophila hydei] | 1 | 4.79 |
| 6b07 | | CR383676.21 | 1.00E-32 | Zebrafish DNA sequence from clone CH211-276I12 in linkage group 14 | 2 | 4.74 |
| 8g1 | | AJ293391.1 | 4.00E-18 | Homo sapiens mRNA differentially expressed in malignant melanoma, clone MM J9 | 5 | 4.60 |
| 47c10 | | ABE60902.1 | 4.00E-60 | proteasome activator PA28 subunit [Cyprinus carpio] | 1 | 4.52 |
| 4f11 | | NP_001013473.1 | 9.00E-69 | hypothetical protein LOC541327 [Danio rerio] | 1 | 4.41 |
| 9e08 | | XP_001376099.1 | 1.00E-64 | similar to ribosomal protein S8 [Monodelphis domestica] | 2 | 4.36 |
| 1e02 | | XP_001054789.1 | 2.00E-31 | similar to 60S ribosomal protein L37a [Rattus norvegicus] | 1 | 4.12 |
| 7a04 | | AF322651.2 | 1.00E-18 | Cyprinus carpio isotocin precursor, exons 1, 2 and 3 and complete cds | 1 | 3.94 |
| 8g09 | | NP_998369.1 | 3.00E-59 | hypothetical protein LOC406485 [Danio rerio] | 2 | 3.94 |
| 7d07 | | AM422109.1 | 1.00E-04 | Danio rerio apaf1-like mRNA | 1 | 3.68 |
| 47b8 | | P61368 | 2.00E-67 | 60S ribosomal protein L15 [Carassius auratus] | 3 | 3.63 |
| 6d11 | | BAC53768.1 | 2.00E-78 | invariant chain like protein 2 [Cyprinus carpio] | 1 | 3.61 |
| 9c07 | | NA |  |  | 1 | 3.55 |
| 10c4 | | XP_694118.2 | 3.00E-31 | hypothetical protein [Danio rerio] | 1 | 3.40 |
| 50g1 | | NP_957020.1 | 3.00E-79 | secretogranin V [Danio rerio] | 1 | 3.39 |
| 7f07 | | NA |  |  | 1 | 3.35 |
| 6e04 | | NP_956353.1 | 2.00E-46 | ribosomal protein S3A [Danio rerio] | 1 | 3.29 |
| 3b01 | | NM_001040321.1 | 4.00E-70 | Danio rerio zgc:136559 (zgc:136559) | 1 | 3.27 |
| 4c12 | | AAH49038.1 | 3.00E-69 | Zgc:73149 protein [Danio rerio] | 1 | 3.25 |
| 7e01 | | BAF98660.1 | 2.00E-46 | ribosomal protein L13 [Solea senegalensis] | 1 | 3.23 |
| 35d5 | | NP_001012316.1 | 2.00E-10 | ribosomal protein S24 isoform 1 [Danio rerio] | 1 | 3.18 |
| 69a2 | | NA |  |  | 1 | 3.09 |
| 3e12 | | XP_001506170.1 | 3.00E-43 | similar to 60S ribosomal protein L36 isoform 1 | 2 | 3.07 |
| 2g1 | | ABX72174.1 | 2.00E-43 | cytochrome oxidase subunit II [Cyprinus carpio] | 4 | 3.06 |
| 9d04 | | NP_957146.1 | 1.00E-96 | ribosomal protein S9 [Danio rerio] | 4 | 2.91 |
| 11c2 | | EDL28989.1 | 1.00E-22 | mCG146274 [Mus musculus] | 1 | 2.89 |
| 48e5 | | AAH59530.1 | 8.00E-30 | Ribosomal protein L24 [Danio rerio] | 1 | 2.82 |
| 2g06 | | NP_001103591.1 | 5.00E-75 | Unknown (protein for MGC:171710) [Danio rerio] | 1 | 2.81 |
| 9c08 | | NP_775371.1 | 6.00E-67 | nascent polypeptide-associated complex alpha subunit [Danio rerio] | 3 | 2.73 |
| 7d02 | | AB308069.1 | 5.00E-08 | Hydroides elegans mRNA for putative NADH dehydrogenase 5 | 2 | 2.73 |
| 51e5 | | EAW71418.1 | 8.00E-11 | RNA binding motif protein 8A, isoform CRA_a [Homo sapiens] | 1 | 2.71 |
| 74f9 | | BC067193.1 | e-105 | Danio rerio high-mobility group box 1, mRNA, complete cds | 2 | 2.70 |
| 7a09 | | NA |  |  | 1 | 2.67 |
| 5e04 | | BAF98677.1 | 8.00E-65 | ribosomal protein L27 [Solea senegalensis] | 2 | 2.64 |
| 7b03 | | BAE89681.1 | 5.00E-66 | unnamed protein product [Macaca fascicularis] | 3 | 2.63 |
| 7a11 | | NA |  |  | 1 | 2.61 |
| 61h6 | | BC066752.1 | 7.00E-78 | Danio rerio calmodulin 3b | 3 | 2.61 |
| 3b05 | | XP_855162.1 | 7.00E-39 | similar to U6 snRNA-associated Sm-like protein LSm6 | 1 | 2.59 |
| 6c09 | | BAE97650.1 | 9.00E-58 | NADH dehydrogenase subunit 2 [Cyprinus carpio] | 1 | 2.57 |
| 9e07 | | NA |  |  | 1 | 2.57 |
| 57g6 | | NP_001001590.1 | 1.00E-48 | ribosomal protein L3 [Danio rerio] | 1 | 2.56 |
| 7b07 | | EF112410.1 | 2.00E-35 | Cirrhina molitorella clone HLJLY-12 microsatellite sequence | 2 | 2.56 |
| 3d04 | | NP_998373.1 | 2.00E-67 | ribosomal protein L19 [Danio rerio] | 2 | 2.54 |
| 3g10 | | NA |  |  | 1 | 2.52 |
| 11h10 | | YP_913282.1 | 2.00E-64 | cytochrome c oxidase subunit III [Hemibarbus barbus] | 3 | 2.51 |
| 52c12 | | NP_001070200.1 | 1.00E-75 | ribosomal protein S16 [Danio rerio] | 1 | 2.46 |
| 58a12 | | NM_199819.1 | 4.00E-11 | Danio rerio phosphoserine aminotransferase 1 (psat1) | 1 | 2.45 |
| 62d12 | | AAH56721.1 | 2.00E-63 | Bsg protein [Danio rerio] | 3 | 2.42 |
| 3e06 | | CT737233.17 | 2.00E-34 | Zebrafish DNA sequence from clone DKEYP-87F2 in linkage group 11 | 1 | 2.37 |
| 5c10 | | YP_001654971.1 | 1.00E-48 | ATP synthase F0 subunit 6 [Ctenopharyngodon idella] | 2 | 2.35 |
| 7c10 | | AM497809.1 | 2.00E-07 | Nidula niveotomentosa partial mRNA for putative small monomeric GTPase (smg gene) | 1 | 2.35 |
| 11g11 | | NP_001002487.1 | 2.00E-56 | ribosomal protein L35a [Danio rerio] | 1 | 2.34 |
| 72h7 | | NP_001019593.1 | 2.00E-62 | hypothetical protein LOC554127 [Danio rerio] | 1 | 2.32 |
| 17g6 | | CR854915.21 | 2.00E-07 | Zebrafish DNA sequence from clone DKEY-4G13 in linkage group 13 | 1 | 2.25 |
| 8h07 | | AAI54760.1 | 3.00E-33 | Ribosomal protein L7 [Danio rerio] | 1 | 2.22 |
| 9b1 | | ABF74735.1 | 2.00E-30 | cytochrome b [Cyprinus carpio] | 1 | 2.22 |
| 6d01 | | BX324133.12 | 8.00E-07 | Zebrafish DNA sequence from clone CH211-209H1 in linkage group 10 | 1 | 2.20 |
| 75f6 | NP_001002155.1 | | 3.00E-71 | ribosomal protein L21 [Danio rerio] | 1 | 2.20 |
| 3b11 | NA | |  |  | 1 | 2.20 |
| 64e3 | | XP_688879.2 | e-117 | hypothetical protein [Danio rerio] | 2 | 2.18 |
| 70a6 | | NA |  |  | 1 | 2.17 |
| 6c06 | | YP_913406.2 | 3.00E-57 | NADH dehydrogenase subunit 1 [Barbus barbus] | 2 | 2.16 |
| 7d10 | | NA |  |  | 1 | 2.13 |
| 9c06 | | NA |  |  | 1 | 2.12 |
| 1b05 | | NP_001035132.1 | 3.00E-57 | novel protein similar to vertebrate ribosomal protein L32 (RPL32) | 2 | 2.11 |
| 7c12 | | CR854890.17 | 5.00E-11 | Zebrafish DNA sequence from clone DKEY-149L16 in linkage group 24 | 1 | 2.08 |
| 69a10 | | NP_001116308.1 | 7.00E-88 | wu:fk52f12 [Danio rerio] | 1 | 2.07 |
| 9a04 | | Q90YT6 | 5.00E-24 | 60S ribosomal protein L32 [Ictalurus punctatus] | 1 | 2.05 |
| 6h02 | | XR_029754.1 | 1.00E-54 | Danio rerio similar to selenoprotein Pa (LOC100002165), mRNA | 1 | 2.03 |
| 6b09 | | XP_001624571.1 | 1.00E-23 | predicted protein [Nematostella vectensis] | 1 | 2.02 |
| 1e03 | | BAF98676.1 | 4.00E-64 | ribosomal protein L26 [Solea senegalensis] | 1 | 2.02 |
| 6b08 | | BX571686.2 | 5.00E-32 | Carp DNA sequence from clone carpf-C114, complete sequence | 1 | 2.00 |
| 11c11 | | AP009047.1 | 0 | Cyprinus carpio mitochondrial DNA, complete genome | 8 | 0.50 |
| 9f3 | | DQ983941.1 | 2.00E-88 | Cyprinus carpio 16S ribosomal RNA gene, mitochondrial | 5 | 0.49 |
| 6c07 | | JC1348 | 3.00E-77 | hypothetical 18K protein - goldfish mitochondrion | 99 | 0.48 |
| 5f05 | | NP_956317.1 | 2.00E-46 | hypothetical protein LOC336637 [Danio rerio] | 1 | 0.48 |
| 4c09 | | XP_001336639.1 | 2.00E-43 | hypothetical protein [Danio rerio] | 5 | 0.48 |
| 8d5 | | NP_001116090.1 | 7.00E-10 | hypothetical protein LOC100142641 [Danio rerio] | 1 | 0.46 |
| 6e08 | | NP_998170.1 | 2.00E-76 | hypothetical protein LOC406278 [Danio rerio] | 1 | 0.45 |
| 55a12 | | BX649452.4 | 2.00E-44 | Zebrafish DNA sequence from clone DKEY-226L10 in linkage group 24 | 1 | 0.45 |
| 68f6 | | NP_956604.1 | 6.00E-32 | hypothetical protein LOC393280 [Danio rerio] | 1 | 0.44 |
| 5e02 | | NA |  |  | 1 | 0.44 |
| 12g1 | | YP_001056750.1 | 9.00E-07 |  | 1 | 0.43 |
| 6a12 | | CR936477.18 | 1.00E-63 | Zebrafish DNA sequence from clone DKEY-152P12 in linkage group 21 | 1 | 0.43 |
| 79c12 | | NP_001017757.1 | 2.00E-22 | hypothetical protein LOC550453 [Danio rerio] | 1 | 0.43 |
| 2c04 | | NP_001002378.1 | 5.00E-86 | ferritin heavy chain [Danio rerio] | 1 | 0.42 |
| 48f8 | | NP_001020624.1 | 1.00E-49 | hypothetical protein LOC322599 [Danio rerio] | 1 | 0.41 |
| 7a06 | | AAH49011.1 | 3.00E-35 | Isocitrate dehydrogenase 3 (NAD+) alpha [Danio rerio] | 1 | 0.41 |
| 6b11 | | DQ674852.1 | 5.00E-16 | Hypophthalmichthys molitrix clone BL132 microsatellite sequence | 1 | 0.40 |
| 73h10 | | CAM14115.1 | e-135 | DEAD (Asp-Glu-Ala-Asp) box polypeptide 39a [Danio rerio] | 1 | 0.39 |
| 15a12 | | NP_001002616.1 | 2.00E-24 | pellino homolog 2 [Danio rerio] | 1 | 0.38 |
| 4b03 | | XP_691929.1 | 3.00E-38 | hypothetical protein [Danio rerio] | 1 | 0.36 |
| 9d10 | | Q9YGK5 | 2.00E-47 | Corticotropin-lipotropin 2 precursor [Cyprinus carpio] | 4 | 0.36 |
| 68b4 | | XP_001331223.1 | 2.00E-43 | hypothetical protein [Danio rerio] | 6 | 0.35 |
| 1d06 | | NA |  |  | 1 | 0.34 |
| 3c02 | | NA |  |  | 1 | 0.33 |
| 74c12 | | CAM15635.1 | 3.00E-65 | novel protein containing zona pellucida-like domains [Danio rerio] | 1 | 0.33 |
| 6d09 | | BC139660.1 | 1.00E-33 | Danio rerio cDNA clone IMAGE:7225631 | 1 | 0.32 |
| 1d08 | | AB028457.1 | 6.00E-60 | Cyprinus carpio mRNA for thymosin beta b, complete cds | 13 | 0.32 |
| 79g4 | | NA |  |  | 1 | 0.29 |
| 6b01 | | X61010.1 | 4.00E-53 | Cyprinus carpio complete mitochondrial genome | 1 | 0.28 |
| 6a08 | | AY423018.1 | 2.00E-74 | Danio rerio RAP1B, member of RAS oncogene family (RAP1B) mRNA | 1 | 0.27 |
| 66b8 | | YP_913434.1 | e-123 | cytochrome c oxidase subunit I [Labeo batesii] | 18 | 0.27 |
| 75d1 | | NP_001003640.1 | 3.00E-18 | hypothetical protein LOC445246 [Danio rerio] | 1 | 0.27 |
| 5g08 | | O13050 | 3.00E-63 | Gonadotropin subunit beta-1 precursor [Cyprinus carpio] | 2 | 0.26 |
| 2f06 | | NA |  |  | 1 | 0.25 |
| 5g04 | | NP_001070129.1 | 8.00E-25 | hypothetical protein LOC767723 [Danio rerio] | 1 | 0.25 |
| 3a11 | | NP_001116093.1 | 2.00E-40 | hypothetical protein LOC100142645 [Danio rerio] | 1 | 0.24 |
| 74f5 | | NP_001093458.1 | 2.00E-95 | hypothetical protein LOC558007 [Danio rerio] | 1 | 0.23 |
| 3b04 | | CU104710.8 | 2.00E-50 | Zebrafish DNA sequence from clone CH73-252G14 in linkage group 2 | 2 | 0.23 |
| 4h05 | | NA |  |  | 1 | 0.23 |
| 48c11 | | NM_200107.1 | 3.00E-30 | Danio rerio zgc:64114 (zgc:64114) | 1 | 0.22 |
| 9c10 | | AAH45838.1 | 1.00E-71 | Ldha protein [Danio rerio] | 1 | 0.22 |
| 74a6 | | XM_001333063.1 | e-103 | Danio rerio similar to vacuolar protein sorting 26 (LOC795515), mRNA | 1 | 0.22 |
| 7b11 | | CR555298.10 | 6.00E-08 | Zebrafish DNA sequence from clone CH211-25D12 in linkage group 11 | 1 | 0.21 |
| 3c07 | | O13269 | 2.00E-10 | Metallothionein-1 (MT-1) (Metallothionein-I) (MT-I) | 1 | 0.21 |
| 2d11 | | NA |  |  | 1 | 0.21 |
| 5g09 | | AM748466.1 | 8.00E-08 | Vigna unguiculata partial mRNA for putative single-stranded nucleic acid binding R3H | 1 | 0.21 |
| 72g7 | | NP_001007363.1 | 6.00E-56 | hypothetical protein LOC492490 [Danio rerio] | 1 | 0.21 |
| 1f05 | | AAB63598.1 | 2.00E-48 | ovulatory protein-2 precursor [Salvelinus fontinalis] | 3 | 0.20 |
| 9c01 | | X51969.1 | 0 | Cyprinus carpio growth hormone gene | 3 | 0.20 |
| 13b7 | | XP_001332706.1 | 7.00E-07 | similar to beta thymosin-like protein isoform 1 [Danio rerio] | 2 | 0.19 |
| 55e8 | | NP_001018386.1 | 4.00E-65 | hypothetical protein LOC553571 [Danio rerio] | 1 | 0.17 |
| 4h03 | | XP_414410.1 | 2.00E-22 | similar to HT021 [Gallus gallus] | 1 | 0.16 |
| 5d12 | | AAO43731.1 | 1.00E-95 | heat shock cognate 70 kDa protein [Carassius auratus gibelio] | 2 | 0.16 |
| 3f04 | | ACB30108.1 | 2.00E-37 | nucleoplasmin [Danio rerio] | 1 | 0.16 |
| 15b10 | | CAQ15440.1 | 4.00E-85 | PTC7 protein phosphatase homolog (S. cerevisiae) [Danio rerio] | 1 | 0.15 |
| 3c04 | | XP_001332082.1 | 5.00E-33 | hypothetical protein [Danio rerio] | 1 | 0.15 |
| 3b03 | | NA |  |  | 1 | 0.15 |
| 79a10 | | XP_685495.1 | 1.00E-06 | hypothetical protein [Danio rerio] | 3 | 0.15 |
| 74g3 | | NA |  |  | 1 | 0.15 |
| 4f12 | | NA |  |  | 1 | 0.14 |
| 4h10 | | NP_956340.1 | 6.00E-67 | ribosomal protein S12 [Danio rerio] | 1 | 0.14 |
| 2f12 | | CR848021.10 | 7.00E-10 | Zebrafish DNA sequence from clone DKEY-219E21 in linkage group 12 | 1 | 0.14 |
| 2h01 | | XP_001345039.1 | 9.00E-25 | hypothetical protein [Danio rerio] | 1 | 0.14 |
| 74a9 | | NP_571648.1 | 5.00E-30 | death associated protein 1b [Danio rerio] | 1 | 0.13 |
| 1f12 | | NA |  |  | 1 | 0.13 |
| 2f08 | | XP_696819.1 | 2.00E-20 | similar to hCG17599 [Danio rerio] | 1 | 0.11 |
| 3f10 | | NP_956597.1 | 1.00E-48 | hypothetical protein LOC393273 [Danio rerio] | 5 | 0.10 |
| 76b5 | | AAA49207.1 | e-113 | cysteine proteinase | 1 | 0.10 |
| 5f04 | | AAI52496.1 | 4.00E-42 | Zgc:153827 protein [Danio rerio] | 1 | 0.10 |
| 5a02 | | XP_684976.1 | 7.00E-22 | hypothetical protein [Danio rerio] | 1 | 0.10 |
| 2h03 | | XP_001338232.1 | 1.00E-50 | hypothetical protein [Danio rerio] | 2 | 0.10 |
| 7e07 | | XM_693720.2 | 4.00E-12 | Danio rerio hypothetical LOC570260 (LOC570260), mRNA | 1 | 0.10 |
| 9d02 | | XP_001333734.1 | 1.00E-48 | similar to MGC82565 protein isoform 1 [Danio rerio] | 1 | 0.10 |
| 3c06 | | AAI22382.1 | 1.00E-20 | Unknown (protein for IMAGE:8109385) [Danio rerio] | 1 | 0.09 |
| 4h01 | | DQ449030.2 | 9.00E-06 | Lycopersicon esculentum GDP-mannose pyrophosphorylase mRNA | 1 | 0.09 |
| 2b10 | | XP_001184408.1 | 1.00E-15 | hypothetical protein [Strongylocentrotus purpuratus] | 2 | 0.09 |
| 4e10 | | NP_001038304.2 | 7.00E-56 | hypothetical protein LOC557741 [Danio rerio] | 4 | 0.09 |
| 4f02 | | XP_706596.1 | 6.00E-17 | hypothetical protein [Danio rerio] | 2 | 0.09 |
| 4c11 | | XP_001203588.1 | 2.00E-19 | hypothetical protein, partial [Strongylocentrotus purpuratus] | 1 | 0.08 |
| 43c9 | | NP_998604.1 | 2.00E-72 | MID1 interacting protein 1 [Danio rerio] | 1 | 0.08 |
| 2g11 | | AAD23572.1 | 3.00E-23 | fertilization envelope outer layer protein [Cyprinus carpio] | 1 | 0.08 |
| 3f03 | | NP_851295.1 | 1.00E-47 | claudin d [Danio rerio] | 1 | 0.08 |
| 1d02 | | AAH83463.1 | 3.00E-54 | H1m protein [Danio rerio] | 1 | 0.08 |
| 7c01 | | NP_956002.1 | e-105 | basic leucine zipper and W2 domains 1 [Danio rerio] | 5 | 0.08 |
| 1b02 | | NA |  |  | 1 | 0.08 |
| 61b7 | | CAA88735.1 | e-142 | ZP3 [Cyprinus carpio] | 35 | 0.07 |
| 5d09 | | XP_001344249.1 | 2.00E-38 | hypothetical protein [Danio rerio] | 1 | 0.07 |
| 4f07 | | XP_691720.2 | 1.00E-43 | similar to KAT protein [Danio rerio] | 1 | 0.07 |
| 2e04 | | NP_775358.1 | 6.00E-08 | small nuclear ribonucleoprotein polypeptide C [Danio rerio] | 5 | 0.07 |
| 5c1 | | XP_001639183.1 | 6.00E-24 | predicted protein [Nematostella vectensis] | 5 | 0.06 |
| 4d02 | | NA |  |  | 1 | 0.06 |
| 4b04 | | NP_001092706.1 | 1.00E-08 | hypothetical protein LOC504078 [Danio rerio] | 1 | 0.06 |
| 2g12 | | BX465837.11 | 2.00E-25 | Zebrafish DNA sequence from clone DKEY-27C15 in linkage group 19 | 1 | 0.06 |
| 3e11 | | NA |  |  | 1 | 0.05 |
| 74e6 | | NP_001073645.1 | 6.00E-84 | hypothetical protein LOC558918 [Danio rerio] | 1 | 0.05 |
| 4h08 | | NP_922919.1 | 1.00E-34 | siaz-interacting nuclear protein [Danio rerio] | 1 | 0.05 |
| 5f01 | | CAQ14645.1 | e-105 | novel protein with a zona pellucida-like domain [Danio rerio] | 1 | 0.05 |
| 4b09 | | NA |  |  | 1 | 0.05 |
| 1e01 | | XP_698633.2 | 2.00E-30 | similar to extracellular superoxide dismutase [Danio rerio] | 2 | 0.04 |
| 2d10 | | P35481 | 6.00E-52 | Cystatin precursor (Ovarian cystatin) (P12) | 207 | 0.04 |
| 2h10 | | AF249875.1 | e-126 | Cyprinus carpio metallothionein II mRNA, complete cds | 5 | 0.04 |
| 2h04 | | XP_001338431.1 | 5.00E-14 | hypothetical protein [Danio rerio] | 1 | 0.04 |
| 5b01 | | XP_392313.1 | 5.00E-62 | similar to Tubulin at 56D CG9277-PB, isoform B [Apis mellifera] | 2 | 0.04 |
| 2a10 | | XP_697505.2 | 1.00E-42 | similar to serine proteinase inhibitor, clade B, member 1 [Danio rerio] | 1 | 0.04 |
| 2g09 | | AAK01371.1 | 7.00E-61 | histone H2A [Carassius auratus] | 2 | 0.04 |
| 1e12 | | XP_696423.1 | 1.00E-14 | hypothetical protein [Danio rerio] | 1 | 0.03 |
| 74b8 | | AAO17737.1 | 7.00E-22 | tissue inhibitor of metalloproteinases 4 [Takifugu rubripes] | 2 | 0.03 |
| 2c12 | | NP_001082820.1 | 9.00E-44 | S100 calcium binding protein A1 [Danio rerio] | 3 | 0.03 |
| 10d1 | | ABV59078.1 | 5.00E-37 | cathepsin L [Lates calcarifer] | 1 | 0.03 |
| 74b5 | | NP_937754.1 | e-100 | B-cell translocation gene 4 [Danio rerio] | 1 | 0.03 |
| 55h1 | | XP_692703.2 | 2.00E-88 | hypothetical protein [Danio rerio] | 2 | 0.03 |
| 3c08 | | BAB69039.1 | 8.00E-55 | pentraxin [Cyprinus carpio] | 3 | 0.02 |
| 4a05 | | NP_898894.2 | 3.00E-52 | linker histone H1M [Danio rerio] | 2 | 0.01 |
| 3e08 | | BAA95671.1 | 6.00E-80 | C-type lectin [Cyprinus carpio] | 43 | 0.01 |
| 5a03 | | CR381591.9 | 2.00E-19 | Zebrafish DNA sequence from clone CH211-177N5 in linkage group 20 | 3 | 0.01 |
| 3c10 | | NP_001103303.1 | 2.00E-27 | zona pellucida glycoprotein 3-like [Danio rerio] | 2 | 0.01 |
| 61c7 | | XP_001331507.1 | 1.00E-77 | geminin | 1 | 0.01 |
| 2f11 | | XP_693859.1 | 4.00E-37 | hypothetical protein [Danio rerio] | 1 | 0.01 |
| 64a2 | | CAI11854.1 | 3.00E-78 | ATP synthase, H+ transporting, mitochondrial F1 complex, gamma polypeptide 1 | 1 | 0.01 |
| 3b07 | | CAA96572.1 | e-141 | ZP2 [Cyprinus carpio] | 24 | 0.01 |
