# Supplementary material for: Defining Global Gene Expression Changes of the Hypothalamic-Pituitary-Gonadal Axis in Female sGnRH-Antisense Transgenic Common Carp (Cyprinus carpio)
Source: PLoS One. 2011 Jun 10;6(6):e21057. doi: 10.1371/journal.pone.0021057 (PMC3112210; doi:10.1371/journal.pone.0021057)
Supplement: Table S5 — Primers used to detect 25 chosen genes in real-time quantitative RT-PCR. (DOC) [file pone.0021057.s008.doc]

**Table S5.** Primers used to detect 25 chosen genes in real-time quantitative RT-PCR.

| **Dector** | **Abbreviation** | **Primers (5'–3')** | **Product size (bp)** |
| --- | --- | --- | --- |
| **hypothalamus** |  |  |  |
| Hemoglobin subunit alpha | *hbα* | CAACCAGACCTCCCACAAG | 199 |
|  |  | ATCAGCCCTAAAGCCGA |  |
| NADH dehydrogenase | *nd* | GCACCAGCACAGACAAC | 104 |
|  |  | GCTGACGAAGCATCCGAAA |  |
| Arginine methyltransferase | *prmt* | CTCACTGCCACAAAAGGA | 185 |
|  |  | GTCGAGGTCAACGGTAAA |  |
| MCH 1 precursor | *mch* | CCAATCTGCTGAGGACCC | 132 |
|  |  | CCCTTCTGATGATGGCG |  |
| sGnRH | *sGnRH* | ATGGAGTGGAACGGAAGGT | 169 |
|  |  | CAGACAAATGAGAACAAGAGGAA |  |
| **pituitary** |  |  |  |
| Growth Hormone | *gh* | CTGTCTTTTCTTTTTCCCTCC | 123 |
|  |  | AAATCAGATCCATCCTTTACG |  |
| DEAD (Asp-Glu-Ala-Asp) box polypeptide | *dead* | AACTGAAGGACAGCGAGAAA | 194 |
|  |  | CTGCTGATACCGAGACAACC |  |
| Similar to melanoma inhibitory activity protein | *mia* | TCAACAACACTACTGGGGAAA | 101 |
|  |  | TGAAAGACCGTGGGAACC |  |
| Gonadotropin beta subunit 1 | *fshβ* | TAAACTCATAGGTCTCGTAGGTCCAT | 182 |
|  |  | TCGGCTCACCAATATCTCCAT |  |
| Pyruvate kinase M2 | *pkm2* | CAGTGGTATCAACGCAGAG | 101 |
|  |  | GAATAAAGCGAAATCACATC |  |
| Secretogranin III | *sgIII* | GCGTAGGGTTTGTGGTT | 117 |
|  |  | CTAATGGCTTCTCCTCAGTC |  |
| **Ovary** |  |  |  |
| Cystatin precursor | *cst* | CTTCGGAACGGCACA | 156 |
|  |  | AACAGGCAAAGCAACG |  |
| C-type lectin | *lec* | GCAACTGGTGCTCTAATGA | 155 |
|  |  | GAACAGGATGACTGCGTAT |  |
| B-cell translocation gene 4 | *btg4* | AGTTCAGTGAGTTTGGGCAG | 126 |
|  |  | AACTCTTATGAGGTGGGTATGG |  |
| GABA neurotransmitter transporter 1 | *gnt1* | GCACCAGCACAGACAAC | 102 |
|  |  | GCTGACGAAGCATCCGAAA |  |
| High-mobility group box 1 | *hmgb1* | TAAAATAGGATAACATGGTCATAGC | 228 |
|  |  | CTATTACATTTAGCGTGAAGAAGAT |  |
| Basigin | *bsg* | AGAGGTCTGGTTCTGGTGG | 115 |
|  |  | GAAGCTGTTTGGCGTGATT |  |
| ZP2 | *zp2* | CAGACAGTGTTACTATGGGAGG | 147 |
|  |  | AGGAGCACAAGGTGGGTC |  |
| Gonadotropin beta subunit 1 | *fshβ* | CGGTAATGGAGATATTGGTGAG | 179 |
|  |  | CGTCACGTCTGACAGGAGG |  |
| 40S ribosomal protein S14 | *rps14* | GGAAAAGAAGGAAGAGCAGG | 104 |
|  |  | CAAAGGTGTCGTTGAAGGAT |  |
| S100 calcium binding protein A1 | *s100a1* | CCACCAGCACAACAAAT | 103 |
|  |  | TTATCAGCAAGTAAAGACCC |  |
| ZP3 | *zp3* | CCTTCTTCACCTCCACAT | 127 |
|  |  | AAGTTTCCCATCGTTCC |  |
| Superoxide dismutase | *sod* | TCCAAAGCCAGAAGTCGT | 174 |
|  |  | CATCGGCAGGCAAACC |  |
| Geminin | *gmnn* | CCCTGTTCAGTGTTCTCC | 107 |
|  |  | CAGCTCCTGTAACTCCTCAT |  |
| Pentraxin | *ptx* | GGTCTCACTGCCTTCTGG | 167 |
|  |  | TTCACATCTGTAACTTCTCCC |  |
